# How does regulating doctors’ admissions affect health expenditures? Evidence from Switzerland

**DOI:** 10.1186/s12913-022-07735-7

**Published:** 2022-04-13

**Authors:** Michel Fuino, Philipp Trein, Joël Wagner

**Affiliations:** 1 Department of Actuarial Science, University of Lausanne, Chamberonne - Extranef, Lausanne, 1015 Switzerland; 2 Department of Political Studies, University of Lausanne, Géopolis, Lausanne, 1015 Switzerland; 3 Swiss Finance Institute, University of Lausanne, Chamberonne - Extranef, Lausanne, 1015 Switzerland

**Keywords:** Healthcare system, Doctors’ admissions, Moratorium, Cost containment

## Abstract

**Background:**

Cost containment is a major issue for health policy, in many countries. Policymakers have used various measures to deal with this problem. In Switzerland, the national parliament and subnational (cantonal) governments have used moratoriums to limit the admission of specialist doctors and general practitioners.

**Methods:**

We analyze the impact of these regulations on the number of doctors billing in free practice and on the health costs created by medical practice based on records from the data pool of Swiss health insurers (SASIS) from 2007 to 2018 using interrupted time series and difference-in-differences models.

**Results:**

We demonstrate that the removal of the national moratorium in 2012 increased the number of doctors, but did not augment significantly the direct health costs produced by independent doctors. Furthermore, the reintroduction of regulations at the cantonal level in 2013 and 2014 decreased the number of doctors billing in free practice but, again, did not affect direct health costs.

**Conclusions:**

Our findings suggest that regulating healthcare supply through a moratorium on doctors’ admissions does not directly contribute to limiting the increase in health expenditures.

**Supplementary Information:**

The online version contains supplementary material available at (10.1186/s12913-022-07735-7).

## Introduction

A major challenge for policymakers around the world is to keep the cost of healthcare under control while maintaining the quality and affordability of services [1]. In the literature, there are different explanations for rising healthcare expenditures. One potential reason for rising costs is “supplier-induced demand”, which claims that a higher number of medical doctors increases health services and therefore health expenditures [2–6]. Another explanation for higher costs is “supply-sensitive care”, which points out that the more healthcare services are available the more patients will consume them [7, 8]. To deal with these problems, policymakers can adjust regulations, for example through wage and price control [9, 10], introducing Diagnose Related Groups that enhance transparency and efficiency of the billing of services [11], as well as bringing forward global budgeting systems that replace fee for service payment systems [12–14].

This study contributes to the literature on regulatory adjustments for cost control in ambulatory healthcare by analysing the impact of a moratorium on new admissions of doctors in Switzerland. Similar to the United States and to other countries [15, 16], Switzerland has faced a steep increase in health expenditures during the last decades. Thus, an important goal of health policy reforms has been to get costs under control [17, 18]. Although researchers consider the Swiss health system to produce high quality care services, rising health costs are of concern and create pressure for reforms [19–21]. To deal with increasing costs, Swiss national and subnational (cantonal) governments have limited the admission of new doctors to practice through regulations. On the one hand, it is plausible to implement such a policy in Switzerland. Fees for services from medical practitioners provide attractive conditions for healthcare providers compared to other European countries, particularly for specialists, and the country’s integration in the European market allows the free movement of persons [22]. On the other hand, such hardball supply-side regulations can have unintended consequences, for example a shortage of specialists and therefore a reduced quality of care. Nevertheless, we know little about the consequences of limiting doctors’ admissions for health costs. In the past, the Australian government used a moratorium to regulate the regional distribution of doctors [23, 24]. While the policy was effective, medical practitioners were highly dissatisfied with it [25]. Previous evidence from Switzerland suggests that lifting the moratorium actually reduces the number of doctors and limits the cost of healthcare [26].

In this article, we deepen the analysis of the Swiss case by using time series data at the cantonal level as well as interrupted time series (ITS) and difference-in-differences (DID) models. Notably, we assess the net impact that the moratorium banning the admission of doctors has (a) on the number of doctors billing in free practice and (b) on direct health costs created by independent doctors, notably medical visits. We use original fine-grained monthly data to measure the development of doctors’ admissions and health expenditures to examine how the termination of the national moratorium and the reintroduction of moratoriums in some cantons have impacted the admission of specialist doctors (SP) and general practitioners (GP), as well as on the related health costs. Our analysis shows that the deregulation of admissions augments the number of newly admitted medical practitioners, however, it does not increase health expenditures from billings of individual doctors. Furthermore, the re-introduction of the moratorium at the cantonal level diminishes medical admissions but without reducing health cost increases. Our results are robust to alternative estimation strategies.

These findings have important policy implications for and beyond Switzerland. Our results suggest that regulating the supply side of healthcare through the admission of doctors affects the number of newly admitted practitioners. Nevertheless, these measures do not reduce health expenditures from services billed by doctors. Yet, regulating the admission of new doctors neither increases such health costs. Thus, we conclude that regulating medical doctors’ admissions is an effective policy instrument to steer the number of healthcare professionals without creating additional health expenditures, however, the instrument does not contribute to reducing health costs.

## Theory and background of the swiss health system

### Theoretical expectations

This article builds on two empirical findings in the literature. First, there is some evidence that regulating medical doctors’ admissions limits the number of providers [23–26]. Second, the paper harks back to research on cost containment in healthcare which has been prolix on the lack of impact of provider regulations on healthcare costs. A recent literature review on the topic summarizes different measures used by governments to keep healthcare costs under control. Therein, the authors do not find evidence for effectiveness regarding 21 out of 41 containment policies. “Policies most often evaluated were payment reforms (10 studies), managed care (8 studies) and cost sharing (6 studies)” [27, p. 71]. Further, they do not report evidence that regulations limiting the number of healthcare providers contribute to cost containment. Another study by the OECD also suggests that measures, “restricting the supply of health professionals have proven ineffective in containing overall health expenditures”; notably, limiting the number of providers reduces competition and increases spending [10, pp. 4-5].

According to the literature, there are different explanations for why regulating the admission of doctors might impact health expenditures, or, in other words, why the density of healthcare providers affects health costs [28–30]. One prominent explanation for this logic is called supplier-induced demand. According to this theory, healthcare providers with an independent business have – under certain conditions – an incentive to augment the number of services they provide as they exploit patients’ dependence on providers’ advice for choosing whether they should buy healthcare services [2–5]. Because of the increase in their own ranks and thus competition, physicians will assume a decrease in patient demand. Supplier-induced demand postulates that physicians will exploit the conflict of interest between their roles as agents of the patient and business owners, and inform patients in a way that leads them to consuming more healthcare services than necessary. Nevertheless, in order for this argument to hold, all patients need to be fully insured and fees for healthcare services are endogenously fixed [31]. Under these conditions, the theory predicts that a higher number of healthcare providers should result in more health expenditures [6].

Nevertheless, there are other explanations for the potential connection between a larger number of physicians and higher health expenditures. For example, the correlation between more doctors and higher health costs could be caused by a pre-existing unsatisfied demand for healthcare services and a higher number of doctors might reduce access costs for patients. Furthermore, causality could be reversed as newly admitted physicians select themselves into regions with already high levels of demand [31]. Researchers have referred to the argument that patients rather than doctors drive health expenditures as supply-sensitive care. This approach holds that the more services are available the more patients will consume them, especially if they suffer from chronic diseases. In this scenario, patients rather than doctors drive service usage and thus healthcare costs [7, 8].

Empirical evidence has supported the correlation between higher numbers of healthcare providers and health expenditures and have shown that there is an effect on income for doctors. Nevertheless, the above-discussed alternative explanations cannot be completely excluded [31]. Consequently, this literature has implications for how the moratorium on doctors’ admissions should impact healthcare costs: research on supplier-induced demand implies that the reduction of practitioners through a moratorium should not decrease (or might even increase) healthcare costs as fewer doctors will exploit their market position and extend services. The reform might then drive healthcare costs. According to this logic, the policy would have the opposite effect as intended. Contrariwise, scholarship on supply-sensitive care implies that the reduction of supply through fewer providers should diminish health costs because patients consume less services. Nevertheless, it is important to note that we do not aim at engaging in a causal analysis of specific hypotheses, but we start from these broad expectations to set up a descriptive analysis.

### Doctors’ admissions and cost containment in Switzerland

This study focuses on Switzerland. The Swiss health system is organized in a decentralized fashion [32]. Historically, the organization of public health and prevention as well as the regulation, financing and provision of healthcare were a cantonal responsibility and the transfer of competencies to the federal government happened gradually [18]. In 1996, the Swiss health insurance law (KVG) entered into force, regulating the market for private health insurers and creating a unified health insurance, including an obligation for every resident to have a health insurance. One of the initial goals of the reform was to get the rising health expenditures under control [33]. In Switzerland, every resident must have a basic health insurance; insurers must accept everyone in their basic health insurance plan regardless of pre-existing conditions. This insurance covers inpatient and outpatient care as well as prescription drugs. Patients can choose different plans, as they are free to select healthcare providers (free selection of doctors, family doctor model, health centre model (HMO), Telmed model). Over the last decades, managed care plans (health insurance plans that reduce to some extent the freedom of choice for patients) have become the most important health insurance plan [34]. In addition, individuals need to pick a yearly amount of out-of-pocket payments for their health insurance plan deductibles (up to CHF 2 500 per year). Patients have to pay co-payments that amount up to 10 percent of the treatment cost, within a limit of CHF 700 per year. Furthermore, patients have to pay 20 percent in co-payments on medication as well as CHF 15 per day for hospital visits [34].

In the Swiss health system, prices for services are fixed. Ambulatory healthcare services are paid for by the tariff system for ambulatory care whereas care in hospitals is reimbursed through a different payment system (SWISS DRG). Doctors are self-employed and use TARMED to bill the services they provide in their ambulatory practice. In addition, doctors – especially if they have a specialist qualification – can also practice in hospitals. Insurers and providers negotiate prices in the TARMED system. Since 2013, the federal government can adjust the prices in TARMED if health insurers and doctors fail to adjust prices [34]. The federal government has conducted such adjustments in 2014 and 2017 [35]. Outpatient care is paid for through contributions from health insurers whereas cantons and health insurers share the cost for inpatient care. To govern ambulatory care, cantons can adjust the number of providers [34].

Since the introduction of the KVG by the Federal Assembly [36], healthcare expenditures in Switzerland have more than doubled. The Federal Statistical Office reports that the total cost increased from CHF 39.1 bn. in 1996 to CHF 80.5 bn. in 2016 representing an annual increase of approximately 4%. Swiss health expenditures are the highest in Europe, both in absolute terms per person (and purchasing power parity) and as a percentage of GDP [21]. This context has yielded an intense political and scholarly debate on the burden of these costs for households. Many factors influence the growth of health expenditures [37]. For example, spending has increased due to the decentralization of skills and financing [30], regional differences in health good consumption patterns [20, 38–40], the size of the economy [41], unemployment [42], and population aging [30]. Regarding the above-mentioned theoretical background, scholars have pointed out that Switzerland potentially faces a problem of a supplier-induced demand, as doctors can impact the quantity of healthcare services [29, 30].

To deal with this problem, the Swiss Parliament decided to tackle increasing health expenditures by reducing the density of service providers [43]. Precisely, the parliament introduced paragraph 55a in the KVG, which allows the Swiss government (Federal Council) to regulate providers. This policy change anticipated that the bilateral agreements between Switzerland and the European Union (EU) would result in an augmentation of health expenditures, because economic conditions in Switzerland attract doctors from other EU countries [44]. The Federal Council used the regulation for the first time in July 2002.

**Fig. 1:**
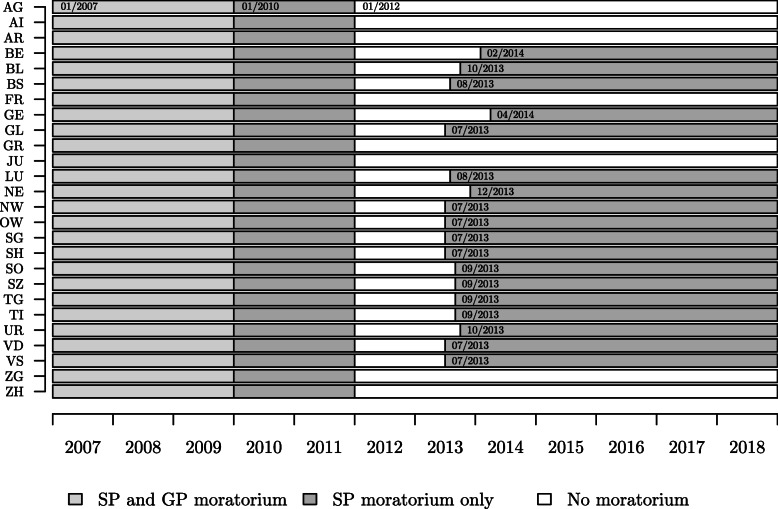
Moratorium periods for SP and GP along the Swiss cantons between 2007 and 2018

In 2007, the Parliament created a moratorium reducing admissions of new doctors [43, 45]. The moratorium distinguished between SP and GP because specialists created much higher health costs [26, 46] and were thus the main target of the policy. Nevertheless, the 26 Swiss cantons are responsible for implementing doctors’ admissions and managing the installation of practitioners in their region [47]. The national moratorium expired on 1st January 2010 for GP and two years later in January 2012 for SP (Fig. 1). In 2013, the Swiss Parliament reintroduced the moratorium [48] due to a sharp increase in the number of doctors, but refused to create a nationwide regulation [49, 50]. Cantons are free to choose whether they implement new rules. Eighteen cantons have reintroduced a moratorium for SP [45]. The federal government defines the main guidelines while the management of admissions is entrusted to the cantons, which follow general principles without distinguishing between national and foreign doctors [48]. Doctors who have worked at a recognized Swiss medical postgraduate institution, for at least three years, are exempted from the moratorium.

The deregulation and re-regulation of doctors’ admissions in Switzerland provide an interesting case study to assess the impact of regulating healthcare providers through a moratorium on the admission of doctors. In the following, we are especially interested in the net effect of these policy changes on the number of doctors and the direct health costs created by these individual practitioners. The combination of deregulation and re-regulation events as well as the Swiss setting with 26 comparable cantonal health systems, which vary in their application of a moratorium, are an interesting case to test the impact of this regulation. Furthermore, the policy is interesting because it affected the migration of professionals to Switzerland. In particular, the removal of the admission ban for SP has increased the immigration of doctors from neighboring countries, whereas the reintroduction of the ban diminished the influx of practitioners [51].

The background of the Swiss case also allows us to study whether there might be some evidence for supplier-induced demand. The population is fully insured, doctors are self-employed and the tariff system TARMED allows them to augment their income through increasing the amount of services provided. Furthermore, doctors can carry out their activities in different practices as well as in hospitals, which allows them to augment their income by providing more services [33, 34]. This corroborates previous research which demonstrated that cantons with a higher number of healthcare practitioners increases the per capita usage of healthcare services and drugs [39].

## Materials and methods

### Dataset on healthcare spending in Switzerland

The data for this study come from the database of Swiss insurers SASIS (see sasis.ch). We can observe the number of doctors billing in free practice and the health costs by specialty in each canton over the period 2007-2018 on a monthly basis (144 months). The records are based on registration numbers identifying independent practitioners and legal entities, which bill medical services to health insurers (“Zahlstellenregister” numbers, ZSR). Our data count the number of unique ZSR billings, in each month and canton by specialty. These data measure the medical claims billed by the ZSR under the KVG. Other healthcare services, such as pharmaceuticals and hospital services are billed using another identifier (see sasis.ch). The benefits paid by all health insurers in Switzerland are recorded by SASIS. Through the ZSR, our data inform about the number of independent SP and GP as well as the direct health costs they create over time. Our data show that the number of doctors billing in free practice and the related health costs have increased for both SP and GP (also see the graphs in the supplementary materials part A). The seasonal effects appear since doctors tend to send their invoices to insurers at the end of each billing period.^1^ We provide further information on the data and descriptive statistics in the supplementary materials (parts A and B).

From the raw data, we create a time-series cross-sectional data set at the level of months and cantons. To operationalize the impact of the moratorium, we define two response (dependent) variables, namely, the number of doctors billing in free practice (*N*_*t*_, related to the aggregated number of ZSR by cantons, see also supplementary materials part A) and health costs (*C*_*t*_) reported during a given month *t*. The independent variables are above all two binary variables identifying the policy change, i.e. the moratorium removal (Rem_*t*_) as well as its later reintroduction (Reint_*t*_) in several cantons. They take the value of 0 before the change and 1 after that. We also account for a variable measuring the number of months since the policy change happened (Month_*t*_). Characterized by a unit increase, this variable takes the value of 1 at the first month of the new policy regulation; it is negative prior the change and positive after that. Finally, we analyze the temporal effect of a policy change by adding the interaction between the number of elapsed months and the policy change dummies (Month_*t*_×Rem_*t*_ respectively Month_*t*_×Reint_*t*_).

To study the removal of the national moratorium for SP in January 2012, we use the 78-months-period from 01/2007 to 06/2013. For comparison purposes, we consider the same period for GP (moratorium removal in January 2010). Concerning the reintroduction of the cantonal moratoriums, we base our study on 84 months of data from 01/2012 to 12/2018, i.e., from the removal date of the national moratorium to the last available record at the moment of data collection. This period includes all canton-specific reintroduction dates (Fig. 1).^2^

### Method of analysis

To analyze the effect of the policy change on both the number of and health costs from doctors, we use interrupted time series models (ITS, e.g., [52, 53]) and a difference-in-differences (DID) model for the reintroduction of the national moratorium, since a control group is available of those cantons that did not reintroduce a moratorium (see, e.g., [54]). Researchers have used both methods to assess the effectiveness of public policies. For example, we find applications for ITS in evaluating speeding limits policies [55] and helmet legislation for cyclists [56]. These models establish a trend in an outcome of interest. This trend is “interrupted by a policy intervention at a particular point in time. The hypothetical scenario under which the intervention had not taken place and the trend continues unchanged (that is: the ‘expected’ trend, in the absence of the intervention, given the pre-existing trend) is referred to as the ‘counterfactual’. This counterfactual scenario provides a comparison for the evaluation of the impact of the intervention by examining any change occurring in the post-intervention period” [52, p. 349]. In implementing this method, it is important to deal with time-varying confounders in the observed outcome, such as seasonality or possible concurrent events [52, pp. 352-354].

In our case, possible concurrent events are the changes in the federal tariff for outpatient medical services, implemented by the federal government in 2014 and at the end of 2017. These events are not concurrent for the removal of the moratorium and for specialists and generalists. Nevertheless, the reform of 2014 increased income possibilities for physicians in outpatient care as of October 2014, in an attempt to shift health cost from the inpatient to the outpatient sector [35]. Our results do not change if we estimate models that control for these potential confounders.

We consider the impact of the removal and reintroduction of the moratoriums on new doctors’ admissions and health costs. Stratified by cantons and type of specialty, we write our model for a response variable *Y*_*t*_∈{*N*_*t*_,*C*_*t*_} (number of doctors billing in free practice, health costs from doctors) and policy intervention *X*_*t*_∈{Rem_*t*_,Reint_*t*_} (moratorium removal, moratorium reintroduction) as follows:

1 $$ \begin{aligned} Y_{t}\!= \beta_{0} + \beta_{1} \, \text{Month}_{t}+ \beta_{2} \, X_{t} + \beta_{3} \, \text{Month}_{t} \times X_{t} + \text{Seasonality}_{t} + \epsilon_{t}.  \end{aligned}  $$

In Eq. 1, *ε*_*t*_ follows a negative binomial distribution when *Y*_*t*_=*N*_*t*_ and follows a log-normal distribution when *Y*_*t*_=*C*_*t*_. In fact, the distributions chosen for both response variables are those that best fit the observations under the Akaike information criterion (AIC, [57]; see also the supplementary materials part C). Since we use the log-link function in the negative binomial and log-normal models, predictions are obtained by taking the exponential of the right hand-side of the equation (1). Further, the coefficient *β*_0_ and the coefficient *β*_1_ are the regression coefficients for the intercept and the elapsed month variable (Month_*t*_). While *β*_0_ represents the baseline level, *β*_1_ indicates the trend before the policy change [52]. After that, *β*_2_ measures the level change or jump due to the policy intervention (*X*_*t*_). However, due to the specification of the Month_*t*_ variable, the interpretation of *β*_2_ must account for the seasonality (cf. next section). The coefficient *β*_3_ specifies the interaction effect (Month_*t*_×*X*_*t*_) by indicating the slope change after the intervention. Regarding health costs from doctors (*Y*_*t*_=*C*_*t*_), we weight the data for price inflation. Finally, in following [58], we use the term Seasonality_*t*_ to control for yearly (e.g., cos(2*π* Month_*t*_/12), sin(2*π* Month_*t*_/12)) and semiannual seasonal effects (cos(2*π* Month_*t*_/6), sin(2*π* Month_*t*_/6)). The introduction of seasonality measures with potential confounders. To account for autocorrelation, we use heteroskedasticity and autocorrelation consistent (HAC) standard errors. For the ITS model, we estimate the models for each canton separately and consider SP apart from GP to rule out small sample effects for the aggregate results.

For the DID model, we include cantonal fixed effects as well as potential confounders into the models (notably nominal GDP, household out-of-pocket payments for health care, unemployment rate and long-term interest rates). The descriptive statistics for these indicators can be found in Appendix B. To draw meaningful conclusions from the DID models, the following conditions need to be fulfilled: Firstly and most importantly, the outcome (in our case the number of doctors and health costs) need to follow parallel trends for the outcome and the control groups. This is no problem in our analysis (cf. Figure 7,supplementary). Secondly, the composition of intervention and comparison groups needs to be stable, which is also the case in our design. Thirdly, the outcome does not need to determine the intervention. Fourth, there should be no spillover effects. In our case, an increasing number of doctors might cause the establishment of a moratorium, but the cantonal governments cannot impact prices which implies that the third assumption holds for health costs. Nevertheless, we cannot completely ignore spillover effects between cantons, yet we are unable to fully control for this problem. Furthermore, it is unlikely that our coefficients are biased since re-introduction of the moratorium at the cantonal level is barely staggered and occurs in a time span of seven month (cf. Fig. 1) [59].

To address in more detail the identification assumptions and additional threats to internal validity of our models, we conduct a number of additional analyses that we report in the supplementary materials of this article. Specifically, to better describe the identification assumption, we provide a goodness-of-fit table (Table 12, supplemen-tary materials), residual analyses (Figure 5, supplementarymaterials), and we validate the linearity assumption upon which our models are based (Figure 6, supplementarymaterials). In order to reproach potential threats to internal validity, we carry out a number of falsification tests, which are reported in Table 13 (supplementary materials). Notably, we modify artificially the interventions at different points in time, use a control group (Table 14, sup-plementary materials), and an out-of-sample test (Figure7, supplementary materials). These tests show that our results are robust against these concerns.

## Results

### Removal of the national moratorium for sP and gP

The results of the regression analysis on SP indicate that removing the moratorium increased the number of doctors billing in free practice in many cantons but did not clearly affect the costs of care (Table 1). Regarding the number of SP billing in free practice (first part of the table), we observe that the values of the intercept and most of the coefficients for the months elapsed are positive and significant, which corresponds to the overall increase observed in the data. The value of the coefficient for the moratorium removal (“Rem.”) is often negative but only statistically significant in the cantons AR, BS, GR, SO, and ZH. This observation indicates an immediate (and counter-intuitive) decrease in the number of SP after the moratorium removal. However, we need to interpret this value considering the interaction term “ Month×Rem.” because we are interested in the comparison of the trends before and after the removal of the moratorium. If we account for the interaction of the trend (running months) and the dummy measuring the removal of the moratorium, we observe a significantly higher growth rate after the removal in 17 cantons.

**Table 1:** Regression results by cantons for the SP national moratorium removal in January 2012

	Number of SP billing in free practice								Health costs from SP							
	Intercept		Month		Rem.		Month×Rem.		Intercept		Month		Rem.		Month×Rem.	
AG	7.946	***	.022	***	−.224	*	.035	***	16.751	***	.050	***	.343		−.020	*
AI	5.429	***	.026	***	−.469		−.015		12.842	***	.030	***	.508		−.038	
AR	6.136	***	.023	***	−.686	***	.018		14.233	***	.022	***	.216		.015	
BE	7.841	***	.017	***	−.278	*	.033	***	17.351	***	.039	***	.570	***	−.014	
BL	7.081	***	.017	***	−.216		.030	**	16.331	***	.024	***	.077		.063	**
BS	6.932	***	.003	*	−.261	**	.049	***	15.918	***	.023	***	.172		.020	
FR	7.435	***	.030	***	−.276	*	.040	***	16.140	***	.049	***	.032		.007	
GE	7.318	***	.008	***	.226	*	.069	***	17.033	***	.028	***	.438		.041	
GL	5.985	***	.031	***	.097		−.030		13.835	***	.027	***	.423		−.094	**
GR	6.997	***	.027	***	−.536	***	.061	***	15.347	***	.034	***	.319		.009	
JU	6.254	***	.022	***	−.028		.042	*	14.518	***	.047	***	−.195		.081	**
LU	7.209	***	−.008		.167		.059	***	16.087	***	.013	***	1.081	***	−.009	
NE	6.654	***	.020	***	−.094		.044	**	15.526	***	.011	*	.773		.014	
NW	6.017	***	.007	*	.150		−.010		13.861	***	.032	***	.184		.037	
OW	5.754	***	.019	***	−.013		.018		13.515	***	.054	***	.739	**	−.029	
SG	7.438	***	.016	***	−.131		.046	***	16.582	***	.036	***	.488	*	−.005	
SH	6.395	***	.033	***	−.366		.049	***	14.856	***	.039	***	.867	*	−.065	***
SO	7.467	***	.023	***	−.256	**	.032	***	16.024	***	.032	***	.380		.002	
SZ	7.227	***	.028	***	−.210		.031	**	15.393	***	.033	***	.596	*	−.028	
TG	7.107	***	.024	***	−.154		.020		15.707	***	.040	***	.772		−.007	
TI	6.934	***	.024	***	−.097		.037	**	16.320	***	.026	***	.313		.023	
UR	5.643	***	.024	***	.183		−.047	**	13.844	***	.082	***	−.030		−.197	***
VD	7.818	***	.024	***	−.010		.023	***	17.149	***	.041	***	.474	*	−.005	
VS	7.430	***	.032	***	−.071		.019		16.086	***	.049	***	.130		−.011	
ZG	6.887	***	.017	***	.050		.029	***	15.254	***	.027	***	.326	*	.006	
ZH	8.175	***	.012	***	−.213	*	.053	***	17.974	***	.037	***	.447		−.039	*
CH									19.499	***	.035	***	.052		.021	

Note: Results are based on 78 months including 60 months before (01/2007–12/2011) and 18 months after (01/2012–06/2013) the removal of the moratorium for SP, see Fig. 1. The displayed values for the coefficients for “Month”, “Rem.” and “ Month×**Rem.**” are multiplied by 10. Values account for the seasonal effect. Significance levels are indicated as follows: * *p*<0.1, ** *p*<0.05, *** *p*<0.01

**Fig. 2:**
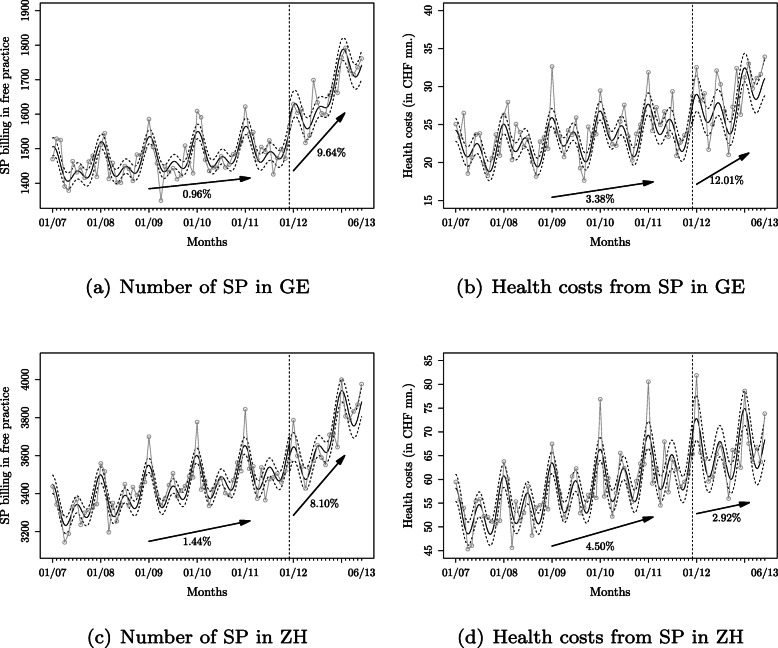
Note: In each graph, the plain curve reports the fit of the regression model and two dashed curves indicate the 95%-confidence interval. The observed data points are illustrated in gray. A vertical dashed line indicates the last date before the removal of the moratorium. Arrows illustrate the estimated annual rate of change in both periods before and after the moratorium. Model predictions for the number of and health costs from SP around the removal of the moratorium in GE and ZH

The graphs (a) and (c) in Fig. 2 illustrate the fit of our regression model in two of the largest cantons, GE and ZH. We provide confidence intervals and compare with the observed data. A vertical dashed line indicates the moratorium removal. Using the reported coefficient values and log-link function of the model, we provide and illustrate the estimated annual rate of change before and after the moratorium removal through “ exp(12*β*_1_)−1” respectively “ exp[12(*β*_1_+*β*_3_)]−1”. For example, in GE, the annual increase in the number of doctors billing in free practice was 0.96% before the moratorium removal and 9.64% after that.

The fit of the model $\hat {Y}_{t}$ presented on the figure can be recovered by applying the estimate of the regression Eq. 1

$$\begin{aligned} \hat{Y}_{t} = & \exp\Big (\hat{\beta}_{0} + \hat{\beta}_{1} \, \text{Month}_{t}+ \hat{\beta}_{2} \, X_{t} + \hat{\beta}_{3} \, \text{Month}_{t} \times X_{t}\\ &+ \hat{\beta}_{4} \cos(2\pi \, \text{Month}_{t}/12)+ \hat{\beta}_{5} \sin(2\pi \, \text{Month}_{t}/12) \\ &+ \hat{\beta}_{6} \cos(2\pi \, \text{Month}_{t}/6)+ \hat{\beta}_{7} \sin(2\pi \, \text{Month}_{t}/6) \Big)+\hat{\epsilon}_{t}, \end{aligned} $$

where the “hat” notation is used to identify the estimates. This equation shows that our focus is on the trend after the removal of the moratorium rather than only on the event.

In the second part of Table 1, we show the results for the SP health costs. Again, both coefficients for the intercept and the elapsed time are significant and positive throughout all cantons. The positive value of the “Month”-coefficient indicates that costs are growing independently of the moratorium question. However, the variables measuring the moratorium removal (“Rem.”) and the interaction (“ Month×Rem.”) are mostly not statistically significant. In BE, LU, and OW, the coefficient for moratorium the increase points at a steep increase of cost but this is not confirmed when we look at the trend in expenditures. Only in two cantons (BL and JU) we observe an increased slope in health costs, while in GL, SH, and UR we find a significant drop in health expenditures after the moratorium removal. In Figs. 2(b,d), we illustrate the results for GE and ZH. Although the value of the slopes increases (decreases) in GE (ZH) after the policy change, the differences are not statistically significant. Overall, this finding suggests that the moratorium removal did not come with an increased expansion of health costs from SP. Consequently, we conclude that eliminating the moratorium on doctors’ admissions for SP has no relevant effect on costs.

The analysis of the removal of the moratorium for GP suggests similar findings (see part C of the supplementary materials for the numerical results). However, when comparing them to the results from SP, the observed changes in the rates of increase before and after the moratorium removal are slightly lower. For example, in GE, the annual change in the number of GP billing in free practice was − 0.96*%* before the moratorium removal and 3.79% after that. This yields a difference of + 4.75 percentage points that can be compared with the 8.68 percentage points increase for SP (cf. above).

### Reintroduction of cantonal moratoriums for sP

We now turn to the assessment of the reintroduction of cantonal moratoriums for SP, which happened in some but not all cantons. We do not consider the effect of the moratorium on GP since the reintroduction targeted SP only. Since only 18 cantons have re-introduced a moratorium, we have a control group available, which allows us to apply a difference-in-differences design. This research design compares the trends before and after moratorium re-introduction in those cantons that actually re-introduced the moratorium with those cantons that did not reintroduce the moratorium. In our analysis, we account for economic (nominal GDP and the unemployment rate), demographic (average age of the population and female-to-male ratio) and health-related (density of hospital beds and household out-of-pocket payment for health care) confounders [38]. We also insert household out-of-pocket payments for health care to control for the demand of particularly expensive treatments.

The findings of the difference-in-differences models show that the reintroduction of the moratorium did not significantly increase the number of practitioners compared to the cantons where no moratorium was reintroduced (Table 2). In other words, the trend in SP admitted for practice does not decrease after the moratorium was introduced. The results are the same for the health costs. Overall, reintroducing a moratorium did neither augment nor diminish health expenditures from SP (Fig. 2).

**Table 2:** Difference-in-differences (DID) model results for the SP national moratorium reintroduction. Coefficients of control variables (nominal GDP, household out-of-pocket payments for health care, unemployment rate, average age of the population, female-to-male ratio and density of hospital beds) are not shown. Result for numbers, significant confounders are nominal GDP, unemployment rate, mean age and density of beds. Results for health costs, significant confounders are unemployment rate, mean age, and the female to male ratio

Intercept		Month		Reint.		Month×Reint.
*Results for number of SP billing in free practice*						
4.362	***	.002	***	−.522	***	−.000
*Results for health costs from SP*						
19.791	***	.002	**	−.603	***	.000

To further examine the re-introduction of the moratorium at the cantonal level, we turn again to ITS models. The results presented in Table 3 show that the reintroduction of the moratorium decreased the number of specialists billing in free practice in most cantons, especially in border cantons (BL, BS, GE, SG, TG, TI, and VD) as well as in cantons with university hospitals (BS, BE, GE, VD). Positive and statistically significant values for the intercept and time variables demonstrate the increase in the number of doctors that is unrelated to the policy change. We observe that 11 out of 18 results for the “Reint.” coefficients are positive and significant. This result entails that a positive jump occurred in the number of SP billing in free practice soon after the reintroduction of the cantonal moratorium. While this result is somewhat curious given that the moratorium restricts admissions, the reader should keep in mind that the number of SP billing in free practice slightly diverges from the number of admissions. As mentioned in [26], just some months before the moratorium, Switzerland delivered five times more admissions to practice when compared to the 2011 average. After this date, doctors have to apply for a ZSR number which takes up to five weeks (cf. SASIS) explaining the jump observed in ZSR billing numbers at moratorium inception. The negative values of the interaction term coefficient indicate how the moratorium policy slowed down the growth in the number of SP billing in free practice. We find the strongest effect for the canton of GE (–0.051, yielding a decrease of the slope by 6 percentage points), which can be explained to the reduced influx of SP from abroad.

**Table 3:** Regression results for the SP moratorium reintroduction in 18 cantons

	Number of SP billing in free practice								Health costs from SP							
	Intercept		Month		Reint.		Month×Reint.		Intercept		Month		Reint.		Month×Reint.	
BE	7.954	***	.059	***	.031		−.038	***	17.523	***	.065	***	.181		−.031	*
BL	7.165	***	.053	***	.315	***	−.035	***	16.526	***	.101	***	−.003		−.077	***
BS	7.013	***	.057	***	.278	*	−.043	***	16.052	***	.086	***	−.128		−.065	**
GE	7.528	***	.065	***	.014		−.051	***	17.244	***	.066	***	.343		−.036	**
GL	5.988	***	−.008		.926	***	.036	***	13.764	***	−.039		1.521	***	.064	**
LU	7.322	***	.049	***	.480	***	−.019	**	16.226	***	.037		.404		.002	
NE	6.758	***	.043	**	.466	**	−.021		15.670	***	.043		1.078	***	.019	
NW	6.021	***	−.011		.646	***	.041	***	13.998	***	.078	***	−.350		−.040	
OW	5.812	***	.028		.472	*	.002		13.618	***	.027		.240		.028	
SG	7.531	***	.056	***	.533	***	−.034	***	16.688	***	.048	***	.952	***	−.020	
SH	6.494	***	.070	***	.394	**	−.035	***	14.888	***	−.020		.745	*	.057	**
SO	7.539	***	.045	***	.484	***	−.025	***	16.137	***	.052	***	.566	*	−.034	*
SZ	7.319	***	.053	***	.312	**	−.022	**	15.479	***	.030		.631	*	.012	
TG	7.193	***	.053	***	.541	***	−.025	**	15.877	***	.066	***	.750	**	−.020	
TI	7.041	***	.055	***	.565	***	−.027	***	16.459	***	.066	***	.555	**	−.012	
UR	5.647	***	−.003		.424		.035	*	13.641	***	−.076	***	−.039		.072	***
VD	7.900	***	.045	***	.311	***	−.018		17.263	***	.049	**	.282		.003	
VS	7.511	***	.046	***	.120		−.010		16.168	***	.055	**	.326		−.022	

Note: Results are based on 84 months from 01/2012 to 12/2018 including the canton-specific reintroduction dates of the moratorium for SP, see Fig. 1. The displayed values for the coefficients for “Month”, “Reint.” and “ Month×**Reint.**” are multiplied by 10. Values account for the seasonal effect. Significance levels are indicated as follows: * *p*<0.1, ** *p*<0.05, *** *p*<0.01

We also examine the impact of the policy change on costs, which is shown in the right part of Table 3. Overall, the regression analyses do not indicate a statistically significant reduction of the direct health costs produced by doctors because of the reintroduction of the moratorium. Again, some cantons (GL, NE, SG, TG, TI) show a positive jump in health expenditures at the removal of moratorium. Nevertheless, if we look at the interaction of the trend and the removal variable, there is no clear result. Overall, reintroducing the moratorium on SP admission did neither increase nor decrease health expenditures. There are, however, interesting differences between cantons. In BL, BS, and GE the reintroduction resulted in a decrease in expenditures. These are border cantons with a proximity to university hospitals. Contrariwise, in GL, SH, UR, the reintroduction of the moratorium augmented health costs. These are small cantons without large urban centers and university hospitals. This result suggests that the effects of the moratorium reintroduction differ between regions. Border cantons with large hospitals experience a decline in costs due to the reduced supply with healthcare services as explained by supply-sensitive-care. Specialists from abroad who work in hospitals refrain from also opening a private practice. In smaller and urbanized cantons without a large hospital the results show an increase in costs after a re-introduction of the moratorium. This logic could be explained with supplier-induced-demand. Established practitioners might have exploited their position and increased the offer in services.

In the supplementary materials, we provide additional analyses, such as the effect of reintroduction by specialty. Furthermore, we present additional robustness tests, notably the analyses of health costs weighted by population size.

## Discussion

Our results suggest that, overall, the removal and reintroduction of the moratorium impacted on the number of doctors billing in free practice, but not on the overall related health costs. These results are different from the findings by [26] who have pointed out that lifting the moratorium increased the number of specialist doctors, while, however, the number of GPs remained rather stable. Their study also suggests that regarding specialists, health costs increased along with the number of doctors. The authors attribute this result to a threshold effect. Nevertheless, the results of this study indicate that consultations per person increased moderately before and after moratorium removal [26]. The results of our analysis indicate that lifting the moratorium increased the number of specialist doctors whereas its re-introduction decreased the number of specialists. At the same time, we show that removing the moratorium for GPs increased the number of doctors in some cantons (9 out of 26). Nevertheless, our findings indicate that lifting the moratorium did not increase the trend of health cost whereas its re-introduction has different effects between cantons – in some health cost decreased – but we do not find an overall effect for Switzerland.

Our findings differ from the previous study because we compare the trends before and after moratorium removal using ITS and DID estimates and also focus on trends at the cantonal level. This method allows us to make more robust estimates that include a time trend variable in the models and analyze differences between cantons. Furthermore, we control for potential confounders over time, such as population growth, nominal GDP, household out-of-pocket payments for health care, unemployment rate, and long-term interest rates.

Therefore, our findings provide robust descriptive evidence that there is neither a clear link between the removal of the moratorium and rising health costs, nor with its re-introduction and a clear decrease in health cost. Although we strengthen our analysis by estimating DID models, the causal implications of our findings need to be interpreted carefully, due to the following reasons.

First, our operationalization measures the number of ZSR billings and not directly the number of doctors. Doctors may practice in several cantons and one doctor may bill services in different specialties. Practitioners receive their ZSR number from SASIS, a private organisation under the control of health insurers. The admission by the cantonal authorities is one of the conditions doctors must fulfill for obtaining a ZSR number. Some doctors obtain a cantonal authorization but apply only later for a ZSR number. Further, our data measure the place where a doctor practices and not the place of admission. Neither can we distinguish between part-time and full-time work. Finally, our data only include costs from independent practitioners and not bills from doctors practicing as employees in an institution (e.g., in a hospital). We cannot include such information as it is not available to us.^3^

Second, we do not have information about doctors practicing in different cantons. In fact, once a practitioner obtains a ZSR number from a canton, the permission is valid everywhere in Switzerland. This permeability among cantons partly jeopardizes the containment effect of the heterogeneous reintroduction of cantonal moratoriums. Another potential issue is that patients can visit doctors in other cantons. This behavior is probably most relevant in small cantons; however, we do not have data available on the movement of patients between cantons. Furthermore, we find that the expansion of the number of doctors billing in free practice decreased also in cantons without moratorium reintroduction (such as AG, FR, and ZH). This observation is puzzling. A possible explanation is that cantons adjusted their practice without formally adapting their policy. Such logic hints at a harmonization of implementation practices, even in the absence of harmonized formal policy changes. In addition, potential applicants, especially from abroad, may have perceived cantonal moratorium re-introductions as a national reintroduction and refrained from applying for positions in Switzerland.

Third, despite the many control variables included in our analysis, we cannot account explicitly for all potentially confounding factors [60], in our analyses. We cannot exclude that other time-varying events impact health costs, due to the absence of a control group and lack of temporal and spatial variance regarding the removal of the moratorium. While population dynamics, e.g. demographic growth and aging, are indirectly included through the time factor, we do not control for regulatory interventions, such as changes in the pricing structure. In addition, the practice of admitting doctors may change without defining new policies, or a new moratorium may be implemented very loosely. Other factors, such as cultural and political characteristics of cantons and their proximity to neighboring countries, could also explain the impact of the moratorium introduction and removal.

These potential caveats do not invalidate the robust statistical evidence that there is no clear link between the moratorium and health cost. Nevertheless, our findings need to interpreted carefully regrading a causal link between taking off the moratorium and changes in health cost, especially regarding conclusions about the absence of supplier-induced demand.

## Conclusion

In this paper, we examine the impact of a moratorium regulating doctors’ admissions on the number of doctors billing in free practice and on health costs in Switzerland. Since the early 2000s, the national government and Federal Parliament have used this policy instrument to better control rising health expenditures by regulating the influx of doctors from other European countries. This regulation expired in 2010 for GP and in 2012 for SP. In mid-2013, some cantons reintroduced a moratorium on doctors’ admissions. We analyze the impact of this deregulation and re-regulation on the number of doctors billing in free practice and on the health costs resulting from free practice. In using original and fine-grained monthly data as well as interrupted time series regression models, we assess how the policy changes affect the number of doctors and health expenditures.

Previous research on the topic has demonstrated that limiting doctors’ admissions through a moratorium reduces the number of practitioners. Concerning the impact of such moratoriums on health costs, the literature is unclear and has argued that a moratorium can either increase or decrease health expenditures. In the Swiss case, scholars have concluded that lifting the moratorium slightly increases cost [26]. Our research confirms previous analyses insofar as we show that limiting doctors’ admissions reduces the actual number of practitioners. However, we highlight that, overall, the abolition and reintroduction of the moratorium has no noteworthy effect on health expenditures. There are some differences between cantons that suggest that the moratorium has different regional effects on health costs.

Our research has important implications for policymakers. From a policy perspective, we suggest that regulatory measures limiting the admission of doctors do not necessarily result in reducing the cost growth and that decision-makers may revert to other instruments to achieve cost containment. Furthermore, particular care should be taken by defining homogeneous measures that apply to the whole country avoiding bias from permeability. From a political perspective, we speculate that the moratorium could be interpreted as a success. On the one hand, lifting the moratorium increased the number of GPs and specialists but it did not increase health expenditures. On the other hand, re-introducing the moratorium reduced the number of physicians but did not affect health expenditures. This link allows politicians to sell the moratorium as a successful instrument for steering the number of health professionals, for example in limiting immigration, without creating additional costs.

Finally, further research should deepen the research on this topic. Specifically, scholars need to engage into further analyses dealing with the causal link between regulations of healthcare supply and health cost, for example by testing more explicitly the predictions from supply-sensitive care and supplier-induced demand theory. Regarding the Swiss health system, such analysis should also account for health costs other than payments to GPs and SP doctors [39]. Furthermore, empirical analyses could engage in models that account even more for seasonality such as ARIMA (Autoregressive Integrated Moving Average) estimators [61].

## Supplementary Information

**Additional file 1** Supplementary materials.

## Data Availability

The data used in this article is not publicly available as it is the property of private organizations (Swiss health insurers). Link to the data: https://www.sasis.ch/. The data can be requested from this organization. No administrative permissions were required to obtain the SASIS data.

## Footnotes

1 Other data sources such as MedReg (medregom.admin.ch) or the statistics of the federal medical association (fmh.ch) would not allow us to link the number of doctors and health costs to the same level of detail, or they are inaccessible to us.

2 Using data from 2007 onward allows us to have a balanced sample of months under a moratorium for SP and the time that passed under the reintroduction of the moratorium in some cantons.

3 We do not weigh the data on billings for the number of doctors at cantonal level as the information is not available on a monthly basis. Nevertheless, information on the number of doctors at the cantonal level can be found here: https://www.fmh.ch/fr/themes/statistique-medicale/statistique-medicale-fmh.cfm$#$i132977.
