# Supplementary material for: How does regulating doctors’ admissions affect health expenditures? Evidence from Switzerland
Source: BMC Health Serv Res. 2022 Apr 13;22:495. doi: 10.1186/s12913-022-07735-7 (PMC9008894; doi:10.1186/s12913-022-07735-7)
Supplement: Supplementary file 1 — Additional file 1 Supplementary materials. [file 12913_2022_7735_MOESM1_ESM.pdf]

RESEARCH

How does regulating doctors' admissions affect health expenditures? Evidence from Switzerland

Michel Fuino[1], Philipp Trein[2]* and Joël Wagner[1,3]

Supplementary materials

This section presents supplementary materials with additional information on our analysis.

A. Comments on the available data

Original data are gathered by matching the ZSR doctor identifiers with the amounts invoiced to health insurers in each month and each canton by specialty as registered in the SASIS data pool. Therefore, this database offers an accurate estimation of the number of doctors billing in free practice by specialty established within one canton and of the related health costs. The available data contain the counts of unique doctor identifiers in each month and in each canton by specialty. The entry reporting the location refers to the canton of work, or, more precisely to the canton where the invoice has been issued. Further, the month of a record represents the month of invoice issuance. Doctors billing services in different cantons have a different ZSR in each canton. Also, one ZSR may bill services in different specialties. Overall, we cannot sum up the cantonal (specialty) counters for the whole Switzerland as we cannot control for duplicate individual doctors among cantons (specialties). Therefore, we only present aggregate figures for the health costs on the level of the whole country (sum of costs from all cantons) but not for the number of doctors (the sum of the cantonal counters would overestimate the true headcount). The health costs from both SP (Figure 1a) and GP (Figure 1b) have increased over time. Seasonal effects appear since doctors tend to send invoices to insurers at the end of each billing period. Further, dashed lines inform about the moratorium removal and reintroduction dates.

Note that the Swiss cantons and their abbreviations are as follows: Aargau (AG), Appenzell Innerrhoden (AI), Appenzell Ausserrhoden (AR), Basel-Landschaft (BL), Basel-Stadt (BS), Bern (BE), Fribourg (FR), Geneva (GE), Glarus (GL), Graubünden (GR), Jura (JU), Lucerne (LU), Neuchâtel (NE), Nidwalden (NW), Obwalden (OW), Schaffhausen (SH), Schwyz (SZ), Solothurn (SO), St. Gallen (SG), Thurgau (TG), Ticino (TI), Uri (UR), Valais (VS), Vaud (VD), Zug (ZG), and Zurich (ZH).

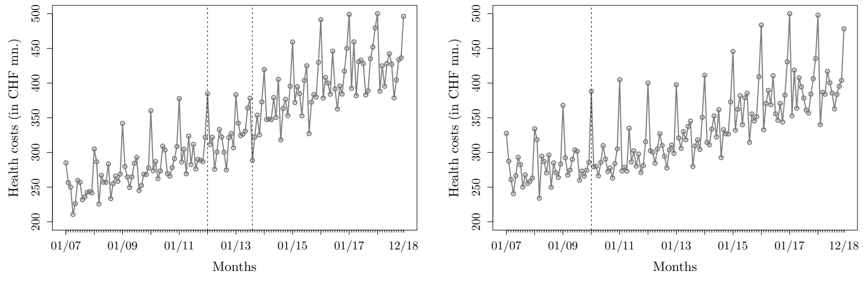

(a) Health costs from SP

(b) Health costs from GP

Note: Vertical dashed lines indicate the removal of the national moratorium for GP (01/2010) respectively for SP (01/2012) and the first reintroduction date of a cantonal moratorium for SP (07/2013).

Figure 1 Health costs from SP and GP in Switzerland from 2007 to 2018.

B. Descriptive statistics

In Table 1, we report the health costs from SP and GP by canton over the period from 2007 to 2018. We observe that the health costs from doctors have strongly increased over the years in each canton. For the overall Switzerland (row "CH"), health expenditures for SP (GP) are of 2 942 CHF mn. (3 252 CHF mn.) in 2007 and increase to 5 154 CHF mn. (4 836 CHF mn.) in 2018. In comparison, the permanent resident population in Switzerland (row "Pop.") is of 7 593 th. and 8 542 th. at the same dates. Therefore, during the 12 years in the observation period, the sum of SP and GP health costs in Switzerland have been multiplied by about 1.6 (9 990/6 194) while the population has only increased by a factor of 1.1 (8 542/7 593). Such outcome shows that the costs by inhabitant have significantly increased over the years.

	2007		2008		2009		2010		2011		2012		2013		2014		2015		2016		2017		2018	
	SP	GP	SP	GP	SP	GP	SP	GP	SP	GP	SP	GP	SP	GP	SP	GP	SP	GP	SP	GP	SP	GP	SP	GP
AG	173	189	191	195	202	199	220	203	228	211	240	219	260	235	281	244	296	268	308	275	323	289	324	294
AI	4	7	4	8	4	8	4	8	5	8	5	8	5	8	5	8	5	9	6	10	6	10	6	10
AR	16	26	17	28	18	29	18	29	19	29	19	29	21	30	23	30	25	32	26	34	26	34	26	36
BE	334	451	366	470	381	476	400	476	422	480	444	491	485	515	512	530	545	579	575	596	600	613	598	619
BL	131	141	142	151	143	152	150	153	154	157	159	160	182	169	187	173	197	185	205	189	216	192	205	197
BS	87	59	92	62	97	67	99	68	101	72	103	75	114	80	115	83	121	89	124	92	129	94	124	96
FR	94	76	103	78	112	81	120	81	122	83	129	88	142	93	150	98	157	112	158	127	169	134	174	140
GE	262	157	278	166	284	170	299	174	310	183	332	188	367	202	386	208	413	228	431	236	454	257	449	251
GL	11	24	11	25	12	26	12	26	12	26	12	26	12	27	14	27	16	29	16	30	16	32	15	31
GR	47	88	50	89	52	89	55	89	57	91	59	90	66	96	70	97	74	105	79	108	79	110	79	110
JU	19	16	21	17	21	17	23	19	25	19	26	20	30	21	32	22	35	24	39	25	41	25	39	25
LU	110	209	115	215	115	221	118	225	122	227	131	235	140	242	149	247	158	263	166	271	176	282	176	289
NE	62	41	68	43	67	41	66	41	70	43	72	45	79	46	88	48	102	53	110	54	115	56	121	56
NW	10	21	11	22	12	22	13	22	13	22	14	22	15	23	15	25	16	26	16	27	18	27	18	29
OW	7	21	8	23	8	23	8	23	9	23	10	23	11	23	11	24	12	25	13	26	14	26	14	26
SG	161	262	168	268	177	275	187	279	198	289	206	295	229	305	247	315	260	328	269	336	277	344	278	347
SH	27	31	30	31	31	31	34	32	34	32	37	33	38	33	39	34	43	36	46	38	48	38	46	40
SO	92	147	100	153	103	155	109	156	111	156	117	157	126	162	137	166	139	177	143	183	147	189	145	188
SZ	49	85	52	88	56	93	57	92	60	93	63	97	66	101	71	105	78	114	81	119	87	123	86	130
TG	65	129	71	133	72	134	77	135	83	136	88	139	98	146	106	149	119	157	126	161	132	165	131	169
TI	130	101	137	102	141	103	148	106	152	112	157	115	175	125	192	131	210	147	226	153	238	157	247	163
UR	8	23	9	22	11	22	11	22	12	22	12	21	11	21	11	21	10	23	10	23	10	25	10	25
VD	271	200	297	212	306	215	331	223	344	230	367	240	393	249	417	257	454	290	489	300	528	307	531	308
VS	91	82	98	91	103	93	112	94	119	100	122	102	131	108	143	115	146	132	144	147	158	149	166	150
ZG	44	51	47	53	48	55	51	57	52	57	54	58	58	61	62	64	69	71	69	74	71	77	71	80
ZH	637	615	678	642	714	660	760	671	784	697	805	739	857	797	943	837	985	899	1 035	954	1 086	1 003	1 075	1 027
CH	2 942	3 252	3 164	3 387	3 290	3 457	3 482	3 504	3 618	3 598	3 783	3 715	4 111	3 918	4 406	4 058	4 685	4 401	4 910	4 588	5 164	4 758	5 154	4 836
Pop.	7 593		7 702		7 786		7 870		7 955		8 039		8 140		8 238		8 327		8 420		8 484		8 542	

Note: Health costs are reported in CHF mn. The row "Pop." indicates the permanent resident population in Switzerland and is expressed in th.

Table 1 Health costs from SP and GP by cantons from 2007 to 2018.

	2007	2008	2009	2010	2011	2012	2013	2014	2015	2016	2017	2018
Nominal GDP (in CHF mn.)	592 442	617 696	607 377	629 325	641 200	648 981	660 649	672 818	675 736	685 441	693 694	719 614
Unemployment rate (in %)	-	-	-	4.8	4.4	4.5	4.7	4.8	4.8	4.9	4.8	4.7
Average age of the population (in years)	40.5	40.7	40.9	41.1	41.2	41.3	41.5	41.8	41.8	41.9	42.1	42.3
Female-to-male ratio (in %)	1.02	1.02	1.02	1.02	1.01	1.01	1.01	1.01	1.01	1.00	1.00	1.00
Households health payments (in CHF)	2 008.0	2 029.1	2 069.3	2 093.9	2 082.2	2 151.3	2 150.0	2 271.2	2 322.3	2 458.0	2 488.3	2 691.3
Number of hospital beds (per 1 000 inhabitants)	4.9	4.9	4.8	4.7	4.6	4.6	4.5	4.3	4.3	4.3	4.2	4.2

Table 2 Selected economic indicators for Switzerland from 2007 to 2018.

	Number of GP billing in free practice				Health costs from GP			
	Intercept	Month	Rem.	Month × Rem.	Intercept	Month	Rem.	Month × Rem.
AG	7.736 ***	.015 ***	.019	.005	16.582 ***	.007	−.039	.031 ***
AI	5.015 ***	.027 **	−.076	−.002	13.389 ***	.021	−.298	−.010
AR	5.946 ***	.017 **	.321 *	−.007	14.639 ***	.017	.138	−.014
BE	7.701 ***	−.000	−.071	.013 **	17.455 ***	.006	−.166	.014
BL	6.921 ***	.007 *	−.048	.005	16.315 ***	.011	−.061	.010
BS	6.837 ***	.016 ***	−1.106 ***	−.015 *	15.504 ***	.037 ***	−.213	.007
FR	7.042 ***	.026 ***	−.187	.008	15.677 ***	.011	−.202	.026 **
GE	7.018 ***	−.008	−.335 *	.039 ***	16.434 ***	.018 **	−.128	.021 *
GL	5.485 ***	.016	−.133	.019	14.551 ***	.017	−.172	−.007
GR	6.752 ***	.014 ***	−.170	.012 *	15.763 ***	−.012	−.021	.032
JU	5.713 ***	−.001	.013	.024 **	14.147 ***	.008	.776	.013
LU	7.053 ***	−.067 ***	.516 *	.081 ***	16.676 ***	.003	.220	.015
NE	6.293 ***	.007	−.114	.007	14.979 ***	−.026	.181	.057 ***
NW	5.825 ***	.021 ***	−.265	−.003	14.348 ***	−.009	.364	.010
OW	5.423 ***	.019 **	.083	.021 *	14.434 ***	.009	−.247	−.002
SG	7.271 ***	.001	.055	.021 ***	16.887 ***	−.002	.352	.020
SH	5.938 ***	.020 ***	−.255	.019 **	14.725 ***	−.007	.287	.014
SO	7.225 ***	.017 ***	−.220	.007	16.330 ***	.005	.063	.001
SZ	6.866 ***	.021 ***	−.192	.016 **	15.806 ***	.015	−.084	.007
TG	6.916 ***	.010 *	−.198	.019 ***	16.167 ***	−.006	.192	.021
TI	6.656 ***	.016 **	−.278	.024 ***	15.912 ***	−.007	.110	.051 ***
UR	5.296 ***	.040 ***	−.826 **	−.017	14.330 ***	−.050 ***	.731	.027
VD	7.488 ***	.021 ***	−.155	.005	16.660 ***	.011	.154	.021
VS	7.054 ***	.014 *	.005	.009	15.845 ***	.034 **	−.286	.004
ZG	6.607 ***	.021 ***	−.025	.003	15.306 ***	.019 *	.009	−.001
ZH	8.057 ***	.009 ***	.017	.000	17.781 ***	.012	−.151	.033 ***
CH					19.434 ***	.007	−.009	.021 *

Note: Results are based on 78 months including 36 months before (01/2007–12/2009) and 42 months after (01/2010–06/2013) the removal of the moratorium for GP, see Figure ??. The displayed values for the coefficients for "Month", "Rem." and "$\text{Month} \times \text{Rem.}$" are multiplied by 10. Values account for the seasonal effect. Significance levels are indicated as follows: * $p < 0.1$, ** $p < 0.05$, *** $p < 0.01$.
Table 3 Regression results by cantons for the GP national moratorium removal in January 2010.

C. Regression results for the removal of the national moratorium for GP

In Table 3 we report our analysis of the removal of the moratorium for GP. The "Month"-coefficient is not always statistically significant meaning an unclear picture regarding the overall growth rate of the number of GP billing in free practice. Additionally, only in few cantons the removal and the interaction term are relevant. The interaction term has a significant positive effect in six cantons, concerning essentially border cantons (GE, LU, JU, SG, TG, TI, and SZ) and cantons with large hospitals (BE, GE). This observation could hint that the moratorium removal influenced the immigration of GP into Switzerland from neighboring countries.

In the right-hand side of Table 3, we report the results estimating the effect on the GP health costs. Apart from the intercept, in most cantons no other coefficient is statistically significant. In particular, the time variable ("Month") demonstrates that health costs remained stable during the whole observation period. Only in NE, TI, and ZH, we find a significantly positive interaction term coefficient. Thus, observations for GP are mostly aligned with findings from SP (Table **??**), indicating that the moratorium removal (alone) did not increase further health expenditures.

D. Reintroduction of cantonal moratoriums for SP

In addition to the results presented in the main text of the article, the representation in Figure 2(a) illustrates that, for GE, before the moratorium reintroduction, a yearly growth of 8.11% in the number of doctors billing in free practice is observed. After the reintroduction, this rate was reduced to 1.56%. In Figure 2(b), the lower slope for costs in GE is not statistically significant (see Table 3 in the main article).

E. Regression results for SP health costs by specialty

In Table 4, we present the regression results for SP health costs by specialty for both the removal and reintroduction of the moratorium. We only report results for

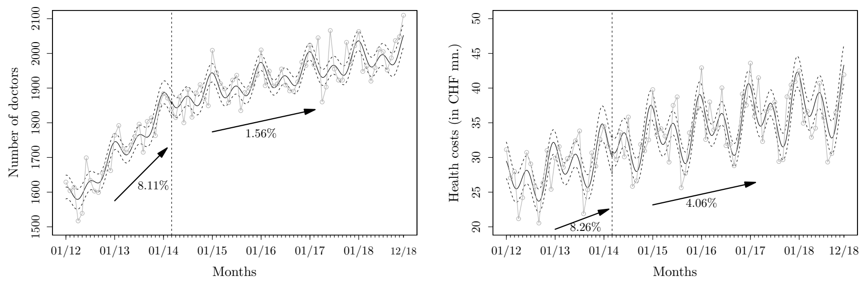

(a) Number of SP in GE (b) Health costs from SP in GE

Note: In each graph, the plain curve reports the fit of the regression model and two dashed curves indicate the 95%-confidence interval. The observed data points are illustrated in gray. A vertical dashed line indicates the last date before the reintroduction of the moratorium. Arrows illustrate the estimated annual rate of change in both periods before and after the moratorium reintroduction.

Figure 2 Model predictions for the number of and health costs from SP for the reintroduction of the cantonal moratorium in GE.

	Removal of the national moratorium for SP								Reintroduction of cantonal moratoriums for SP							
	Intercept		Month		Rem.		Month × Rem.		Intercept		Month		Reint.		Month × Reint.	
Allergology and clinical immunology	14.652	***	.012	**	−.738	**	.046	***	14.496	***	.026	*	.488		−.010	
Anesthesiology	14.977	***	.096	***	−.531		−.003		14.483	***	.103	***	1.886	***	−.055	*
Angiology	15.137	***	.021	***	−.017		.019		14.816	***	.031	*	.047		.020	
Cardiology	16.425	***	.025	***	.051		.004		16.138	***	.055	***	.213		−.003	
Surgery	15.583	***	−.007		.396		.018		15.262	***	.035	**	−.199		−.034	**
Maxillo-facial surgery	13.408	***	.023	***	−.099		.019		12.910	***	.062	**	.031		−.062	**
Orthopaedic surgery, traumatology	15.819	***	.025	***	.394		.009		15.608	***	.066	***	.625		−.032	
Plastic, reconstructive, aesthetic surgery	14.450	***	.059	***	.869	***	.007		14.372	***	.072	***	.424		−.042	*
Dermatology and venereology	16.447	***	.034	***	.562		.003		16.159	***	.051	***	.355		−.010	
Endocrinology and diabetology	15.200	***	.017	***	−.112		.056	***	15.033	***	.052	***	−.204		−.064	***
Gastroenterology	16.489	***	.032	***	.228		.021		16.190	***	.039	**	.936	*	−.015	
Gynecology and obstetrics	17.360	***	.029	***	−.034		−.003		16.997	***	.033	**	.312		−.015	
Hematology	14.516	***	.009		1.046	**	.138	***	14.664	***	.086	***	−.568		−.058	*
Physical medicine and rehabilitation	14.544	***	.053	***	−1.001	***	−.013		14.052	***	.035	**	−1.399	***	−.010	
Physicians, special cases	13.965	***	.003		−2.527	**	−.069		13.129	***	−.070		1.923	**	.063	
Nephrology	15.088	***	−.001		1.920	**	−.115		14.453	***	.009		−.385		.012	
Neurosurgery	13.587	***	.021	***	−.604		.255	*	13.765	***	.182	***	.624		−.171	***
Neurology	15.751	***	.049	***	.268		.011		15.425	***	.081	***	.191		−.022	
Medical oncology	16.615	***	.044	***	−1.025	***	.034		16.261	***	.052	***	−.348		−.027	
Ophthalmology	17.416	***	.048	***	−.006		.050	**	17.222	***	.110	***	.554		−.063	***
Otorhinolaryngology	15.987	***	.009	**	.316		.052	***	15.722	***	.051	***	.122		−.031	***
Pathology	15.355	***	.100	***	.259		−.077	***	15.028	***	.066	***	.463		.056	***
Pneumology	15.539	***	.009	*	.003		.007		15.248	***	.005		.357		.049	**
Psychiatry and psychotherapy	17.659	***	.043	***	.181		.009		17.405	***	.063	***	.294		−.023	**
— of children and adolescents	15.476	***	.040	***	.480		.030		15.241	***	.051	***	.404		.004	
Radiology	17.062	***	.053	***	−.122		.048	**	16.855	***	.071	***	.178		−.055	***
Rheumatology	16.431	***	.029	***	.198		.007		16.033	***	.049	***	.279		−.025	
Urology	15.568	***	.006	*	.030		.010		15.299	***	.039	*	.248		.009	

Note: Results for the moratorium removal are based on 78 months including 60 months before (01/2007–12/2011) and 18 months after (01/2012–06/2013) the removal of the moratorium for SP, see Figure 1 in the main document. Results for the moratorium reintroduction are based on 84 months from 01/2012 to 12/2018 including the canton-specific reintroduction dates. The results concerning the reintroduction of cantonal moratoriums are based on data from the 18 relevant cantons only. The displayed values for the coefficients for "Month", "Rem." and "Month × Rem." respectively "Reint." and "Month × Reint." are multiplied by 10. Values account for the seasonal effect. Significance levels are indicated as follows: * $p < 0.1$, ** $p < 0.05$, *** $p < 0.01$.

Table 4 Regression results for health costs by specialty for the SP national moratorium removal in January 2012 and the SP moratorium reintroduction in 18 cantons.

specialties where on average at least 100 doctors have billed medical services in each month. While the analysis on the removal of the national moratorium uses health costs from all 26 cantons, the study on the reintroduction of cantonal moratoriums only considers the costs from the 18 cantons that have reintroduced the regulation. On the one hand, our results show that removing the moratorium augmented the costs increase from some specialists, such as allergology and clinical immunology, endocrinology and diabetology, and radiology. On the other hand, the reintroduction of the moratorium reduced the costs from these same specialities as well as from other disciplines. Nevertheless, regarding some specialities, such as pathology and

pneumology, costs increased after re-introduction of the moratorium. Overall, we cannot confirm a strong cause-effect relationship of the moratorium policies and the increase in health costs, also because in many specialties particular effects that we do not control for may have a more important impact.

F. Regression results for SP per cantonal population

In the following, we discuss the regression results for SP health costs and for the number of SP billing in free practice when divided by the monthly cantonal population. In Table 5, we present the results for SP health costs divided by the monthly cantonal population on the same period for both the removal and reintroduction of the moratorium. As a robustness test of the results presented in the paper, we highlight here that using the SP heath costs divided by the monthly cantonal population has no significant effect on the results compared to the findings presented in Tables 1 and 2. Our results show that the health costs in most cantons were not affected by the moratorium removal even when accounting for the population size. We come to the same conclusion when considering the moratorium reintroduction effect on the health costs per capita.

	Removal of the national moratorium for SP				Reintroduction of the cantonal moratoriums for SP			
	Intercept	Month	Rem.	Month × Rem.	Intercept	Month	Reint.	Month × Reint.
AG	5.867 ***	.048 ***	−.038	.004				
AI	3.175 ***	.026 ***	.191	−.011				
AR	.890 ***	.010	−.161	.042 **				
BE	4.821 ***	.034 ***	.223	.010	4.981 ***	.058 ***	.141	−.029 *
BL	4.197 ***	.023 ***	−.328	.082 ***	4.373 ***	.093 ***	−.018	−.073 ***
BS	2.115 ***	.019 ***	−.223	.044	2.234 ***	.078 **	−.159	−.063 **
FR	3.575 ***	.034 ***	−.421 *	.031				
GE	3.983 ***	.017 ***	.166	.066 **	4.175 ***	.054 ***	.271	−.034 *
GL	3.260 ***	.022 ***	.009	−.068 *	3.178 ***	−.043	1.494 ***	.065 **
GR	3.170 ***	.029 ***	−.020	.035				
JU	3.354 ***	.045 ***	−.587	.101 **				
LU	3.228 ***	.002	.707 **	.018	3.351 ***	.028	.377	.003
NE	3.462 ***	.007	.374	.036	3.587 ***	.036	1.007 **	.027
NW	3.231 ***	.028 ***	−.207	.061 *	3.356 ***	.072 ***	−.340	−.040
OW	3.018 ***	.042 ***	.443	−.001	3.113 ***	.018	.220	.031
SG	3.489 ***	.028 ***	.116	.021	3.582 ***	.040 **	.926 ***	−.017
SH	3.600 ***	.032 ***	.456	−.041 *	3.614 ***	−.029	.736 *	.059 **
SO	4.114 ***	.022 ***	−.017	.029	4.207 ***	.043 **	.574 *	−.033 *
SZ	2.934 ***	.028 ***	.190	−.003	3.004 ***	.023	.607 *	.012
TG	2.976 ***	.035 ***	.341	.012	3.119 ***	.054 ***	.668 **	−.011
TI	3.877 ***	.014 ***	−.098	.047 *	3.988 ***	.054 ***	.522 *	−.009
UR	3.367 ***	.079 ***	−.443	−.174 ***	3.153 ***	−.080 ***	−.021	.073 ***
VD	4.476 ***	.029 ***	.093	.020	4.566 ***	.036 *	.212	.008
VS	2.580 ***	.033 ***	−.166	.014	2.641 ***	.038	.284	−.016
ZG	3.599 ***	.016 ***	−.094	.032 ***				
ZH	3.820 ***	.024 ***	.086	−.010				

Note: Results for the moratorium removal are based on 78 months including 60 months before (01/2007–12/2011) and 18 months after (01/2012–06/2013) the removal of the moratorium for SP, see Figure 1in the main document. Results for the moratorium reintroduction are based on 84 months from 01/2012 to 12/2018 including the canton-specific reintroduction dates. The results concerning the reintroduction of cantonal moratoriums are based on data from the 18 relevant cantons only. The displayed values for the coefficients for "Month", "Rem." and "Month × Rem." respectively "Reint." and "Month × Reint." are multiplied by 10. Values account for the seasonal effect. Significance levels are indicated as follows: * $p < 0.1$, ** $p < 0.05$, *** $p < 0.01$.

Table 5 Regression results for health costs by cantons for the SP national moratorium removal in January 2012 and the SP moratorium reintroduction in 18 cantons.

Similarly, in Table 6, we present the regression results for the number of SP billing in free practice divided by the monthly cantonal population on the same period for both the removal and reintroduction of the moratorium. Let us also mention that the model specification has been changed since, after division, the outcome variable is no more a counting process that can be modeled by a negative binomial distribution. We modified the previous outcome variable (number of doctors) into a ratio. We ran

the model selection procedure again and found that, among the tested models, the Gaussian specification has the lowest AIC. Further, as seen in the table, coefficient values become small. In this case, our results show that the number of SP billing in free practice costs in most cantons were affected by the moratorium removal even when accounting for the population size. We come to the same conclusion when considering the moratorium reintroduction effect on the number of SP per capita. Such results are coherent with what we observe when solely focusing on the number of SP.

	Removal of the national moratorium for SP				Reintroduction of the cantonal moratoriums for SP			
	Intercept	Month	Rem.	Month × Rem.	Intercept	Month	Reint.	Month × Reint.
AG	.053 ***	.001 ***	−.011	.002 ***				
AI	.014 ***	.000 ***	−.005	−.000				
AR	.001 ***	.000 ***	−.000 ***	.000				
BE	.009 ***	.000 ***	−.002 *	.000 ***	.010 ***	.001 ***	−.000	−.000 ***
BL	.006 ***	.000 ***	−.001	.000 *	.007 ***	.000 ***	.002 ***	−.000 ***
BS	.001 ***	−.000	−.000 **	.000 ***	.001 ***	.000 ***	.000 *	−.000 ***
FR	.006 ***	.000 ***	−.002 **	.000 ***				
GE	.003 ***	−.000	.001 ***	.000 ***	.004 ***	.000 ***	−.000	−.000 ***
GL	.010 ***	.000 ***	.001	−.000	.010 ***	−.000	.009 ***	.000 ***
GR	.006 ***	.000 ***	−.003 ***	.000 ***				
JU	.007 ***	.000 ***	−.000	.000 *				
LU	.004 ***	−.000 ***	.001	.000 ***	.004 ***	.000 ***	.002 ***	−.000 *
NE	.004 ***	.000 ***	−.000	.000 *	.005 ***	.000 **	.002 **	−.000
NW	.010 ***	.000	.001	−.000	.010 ***	−.000	.006 ***	.000 ***
OW	.009 ***	.000 **	.001	.000	.009 ***	.000	.004	.000
SG	.004 ***	.000 ***	−.000	.000 ***	.004 ***	.000 ***	.002 ***	−.000 ***
SH	.008 ***	.000 ***	−.003 *	.000 ***	.008 ***	.000 ***	.003 **	−.000 **
SO	.012 ***	.000 ***	−.003 **	.000 ***	.012 ***	.000 ***	.006 ***	−.000 ***
SZ	.005 ***	.000 ***	−.001	.000 **	.006 ***	.000 ***	.002 *	−.000 **
TG	.004 ***	.000 ***	−.001	.000	.004 ***	.000 ***	.002 **	−.000
TI	.004 ***	.000 ***	−.001	.000 **	.004 ***	.000 ***	.002 ***	−.000 ***
UR	.008 ***	.000 ***	.001	−.000 **	.008 ***	−.000	.003	.000 **
VD	.008 ***	.000 ***	−.000	.000 ***	.008 ***	.000 ***	.002 **	−.000
VS	.002 ***	.000 ***	.000	.000	.002 ***	.000 ***	.000	−.000
ZG	.009 ***	.000 **	.000	.000 ***				
ZH	.003 ***	−.000	−.000	.000 ***				

Note: Results for the moratorium removal are based on 78 months including 60 months before (01/2007–12/2011) and 18 months after (01/2012–06/2013) the removal of the moratorium for SP, see Figure 1 in the main document. Results for the moratorium reintroduction are based on 84 months from 01/2012 to 12/2018 including the canton-specific reintroduction dates. The results concerning the reintroduction of cantonal moratoriums are based on data from the 18 relevant cantons only. The displayed values for the coefficients for "Month", "Rem." and "Month × Rem." respectively "Reint." and "Month × Reint." are multiplied by 10. Values account for the seasonal effect. Significance levels are indicated as follows: * $p < 0.1$, ** $p < 0.05$, *** $p < 0.01$.

Table 6 Regression results for the number of SP billing in free practice for the SP national moratorium removal in January 2012 and the SP moratorium reintroduction in 18 cantons.

G. Difference-in-differences model for the reintroduction of the national moratorium for SP health costs

In the following, we use a difference-in-differences (DID) model for the reintroduction of the national moratorium for SP health costs. The theoretical foundation of this methodology is well documented and can be found in, e.g., [1]. In our approach, we estimate two distinct DID models that we distinguish by a divergence in treatment groups. First, in the model "DID (1)", our treatment group is composed of the cantons that reintroduced the moratorium while our control group consists of cantons that did not reintroduce the moratorium. Second, in the model "DID (2)", the treatment group only accounts for cantons having reintroduced the moratorium in 07/2013 while the control group remains unchanged. In doing so, the second approach allows for having a balanced analysis. The following equation describes both models:

$$\log(C_t) = \beta_0 + \beta_1 \,\mathrm{Month}_t + \beta_2 \,\mathrm{CReint}_t + \beta_3 \,\mathrm{Month}_t \times \mathrm{CReint}_t + \mathrm{Seasonality}_t + \epsilon_t,$$

where the variables C_t, Month_t and ϵ_t are as defined in Section 2.2. The variables CReint_t is a binary indicator taking the value of 1 for cantons having reintroduced the moratorium (treatment group) and 0 otherwise (control group). Further, the variable $\text{Month}_t \times \text{CReint}_t$ represents the interaction effect and is the key measure for evaluating the effect of the moratorium reintroduction on the SP health costs. In our model selection process, we compare this model to similar DID models that include a higher order polynomial form. More precisely, we have compared the AIC values of the presented model (1) with a model adding the quadratic form of the time variable (Month_t^2) and a model adding both its quadratic and cubic forms (i.e. Month_t^2 and Month_t^3). These models have reported AIC values of 7 401, 7 401 and 7 402, respectively, therefore not showing any model improvement. Further, we come to the same conclusion when comparing BIC values. Based on this analysis and the fact that coefficient values and significance levels are quasi identical, we decided to remain with the linear form. We present the results in Table 7.

	Intercept		Month		CReint		Month × CReint
DID (1)	15.420	***	.004	*	.237	*	−.000
DID (2)	15.449	***	.004	*	−.275	*	.000

Note: The notation "DID (1)" refers to a DID model performed on the overall set of cantons while "DID (2)" identifies a balanced DID model where, in the treatment group, solely the cantons having reintroduced the moratorium in 07/2013 are considered.
Table 7 Difference-in-differences (DID) model results for health costs for the SP national moratorium reintroduction.

As a key result, we observe that the variable Month × CReint is not significant, neither in the "DID (1)" nor in the "DID (2)" model. This confirms the absence of an effect of the moratorium reintroduction on health costs. Further, we have performed the DID on the cantons with at least 150'000 inhabitants (BE, GE, ZH, VD and AG) where small sample effects can be excluded. The results are presented in Table 8.

	Intercept		Month		CReint		Month × CReint
DID (1)	17.379	***	.004	***	−.159	*	.000
DID (2)	17.379	***	.004	***	−.209		.001

Note: The notation "DID (1)" refers to a DID model performed on the overall set of cantons while "DID (2)" identifies a balanced DID model where, in the treatment group, solely the cantons having reintroduced the moratorium in 07/2013 are considered.
Table 8 Difference in differences (DID) model results for health costs for the SP national moratorium reintroduction considering AG, BE, GE, VD, ZH.

H. Confidence intervals

In the following, we present the confidence intervals for the regression results presented in the main corpus of the manuscript. This will benefit to the present analysis since it will give some indication whether observed null-effects are stemming from real null-effects or simply from small sample size, i.e. lack of power. In Tables 9 and 10, we present the confidence intervals for the regression results presented in Table 1 and Table 3 in the main document, respectively. For the moratorium removal, the confidence intervals for the number of SP interaction terms (Month × Rem.) exclude, in most cases, the null-effect. The opposite is observed with the

health costs from SP. When considering the moratorium reintroduction, we still observe that most of the interaction terms' confidence intervals exclude the null effect for the number of SP. Again, the opposite is observed for the health costs from SP. These results reflect the conclusions obtained when using p-values.

	Number of SP billing in free practice								Health costs from SP							
	Intercept		Month		Rem.		Month × Rem.		Intercept		Month		Rem.		Month × Rem.	
	Lower	Upper	Lower	Upper	Lower	Upper	Lower	Upper	Lower	Upper	Lower	Upper	Lower	Upper	Lower	Upper
AG	7.932	7.959	.018	.026	−.512	.064	.012	.059	16.716	16.786	.004	.006	−.036	.103	−.007	.004
AI	5.394	5.462	.016	.036	−1.216	.273	−.076	.046	12.783	12.900	.001	.005	−.070	.168	−.014	.006
AR	6.112	6.160	.016	.030	−1.211	−.165	−.025	.061	14.191	14.274	.001	.004	−.066	.107	−.006	.008
BE	7.827	7.855	.013	.021	−.577	.021	.009	.058	17.314	17.387	.003	.005	−.016	.128	−.007	.004
BL	7.066	7.095	.012	.021	−.537	.104	.004	.056	16.289	16.373	.001	.004	−.078	.092	−.000	.013
BS	6.915	6.948	−.002	.008	−.619	.096	.019	.078	15.876	15.959	.001	.004	−.070	.102	−.005	.009
FR	7.420	7.450	.026	.035	−.598	.045	.014	.066	16.102	16.177	.004	.006	−.072	.077	−.005	.007
GE	7.305	7.331	.004	.012	−.051	.503	.046	.091	16.986	17.080	.001	.004	−.049	.135	−.003	.011
GL	5.955	6.015	.022	.040	−.548	.740	−.083	.023	13.781	13.887	.001	.004	−.073	.154	−.019	.000
GR	6.976	7.018	.021	.034	−.995	−.077	.023	.098	15.300	15.393	.002	.005	−.063	.125	−.007	.008
JU	6.229	6.279	.015	.029	−.561	.503	−.001	.085	14.464	14.571	.003	.006	−.127	.085	−.000	.016
LU	7.182	7.235	−.015	−.000	−.406	.740	.012	.106	16.040	16.132	−.000	.003	.016	.198	−.008	.006
NE	6.632	6.676	.013	.026	−.569	.381	.006	.083	15.469	15.581	−.001	.003	−.036	.188	−.008	.010
NW	5.990	6.043	−.001	.015	−.428	.726	−.057	.038	13.810	13.911	.002	.005	−.086	.120	−.004	.012
OW	5.724	5.785	.010	.028	−.673	.645	−.036	.071	13.462	13.566	.004	.007	−.028	.173	−.011	.005
SG	7.422	7.454	.012	.021	−.480	.218	.017	.074	16.533	16.630	.002	.005	−.049	.145	−.008	.007
SH	6.368	6.422	.025	.041	−.949	.216	.001	.096	14.812	14.898	.003	.005	.000	.172	−.013	.000
SO	7.452	7.483	.018	.027	−.592	.081	.005	.060	15.982	16.065	.002	.004	−.047	.121	−.007	.007
SZ	7.210	7.245	.023	.033	−.579	.158	.001	.061	15.342	15.443	.002	.005	−.043	.160	−.011	.005
TG	7.090	7.124	.019	.029	−.527	.218	−.010	.051	15.657	15.756	.002	.006	−.021	.173	−.008	.007
TI	6.912	6.956	.017	.030	−.568	.374	−.002	.075	16.282	16.358	.001	.004	−.046	.107	−.004	.008
UR	5.608	5.678	.014	.035	−.570	.934	−.109	.015	13.786	13.899	.006	.010	−.124	.115	−.030	−.010
VD	7.807	7.829	.021	.028	−.247	.227	.004	.043	17.103	17.194	.003	.005	−.041	.134	−.007	.006
VS	7.409	7.451	.026	.038	−.529	.387	−.018	.057	16.044	16.126	.004	.006	−.069	.093	−.008	.005
ZG	6.863	6.911	.010	.024	−.459	.559	−.013	.070	15.213	15.294	.001	.004	−.051	.114	−.006	.007
ZH	8.164	8.185	.009	.015	−.441	.016	.034	.072	17.937	18.009	.003	.005	−.029	.117	−.010	.002
CH									19.465	19.532	0.025	0.046	−.064	0.073	−0.033	0.075

Note: Results are based on 78 months including 60 months before (01/2007–12/2011) and 18 months after (01/2012–06/2013) the removal of the moratorium for SP, see Figure 1, main document. The notations "Lower" and "Upper" refer to the lower and upper bounds of the 95%-confidence interval
Table 9 Confidence intervals for the regression results presented in Table 1 in the main document.

	Number of SP billing in free practice								Health costs from SP							
	Intercept		Month		Reint.		Month × Reint.		Intercept		Month		Reint.		Month × Reint.	
	Lower	Upper	Lower	Upper	Lower	Upper	Lower	Upper	Lower	Upper	Lower	Upper	Lower	Upper	Lower	Upper
BE	7.932	7.977	.004	.008	−.024	.030	−.005	−.002	17.455	17.588	.002	.011	−.060	.098	−.008	.002
BL	7.140	7.190	.003	.007	.003	.060	−.006	−.001	16.448	16.601	.003	.017	−.086	.088	−.015	−.001
BS	6.986	7.039	.003	.008	−.002	.058	−.007	−.002	15.977	16.123	.001	.016	−.094	.071	−.014	.001
GE	7.510	7.545	.005	.008	−.020	.023	−.006	−.004	17.171	17.314	.002	.012	−.051	.121	−.009	.001
GL	5.936	6.041	−.006	.004	.033	.152	−.002	.009	13.625	13.892	−.017	.009	.010	.303	−.007	.020
LU	7.287	7.357	.002	.008	.008	.088	−.005	.001	16.135	16.313	−.005	.012	−.057	.142	−.008	.009
NE	6.723	6.794	.002	.007	.004	.089	−.005	.001	15.569	15.764	−.003	.012	.000	.220	−.006	.010
NW	5.977	6.065	−.005	.003	.015	.114	−.000	.009	13.916	14.077	−.001	.016	−.124	.057	−.012	.004
OW	5.756	5.868	−.003	.008	−.016	.110	−.005	.006	13.507	13.722	−.008	.014	−.093	.146	−.008	.014
SG	7.503	7.559	.003	.008	.022	.085	−.006	−.001	16.601	16.770	−.004	.014	.004	.190	−.011	.007
SH	6.451	6.537	.003	.011	−.009	.088	−.008	.001	14.793	14.978	−.011	.007	−.026	.179	−.004	.015
SO	7.513	7.565	.002	.007	.019	.078	−.005	−.000	16.063	16.207	−.001	.012	−.023	.139	−.010	.003
SZ	7.286	7.352	.002	.008	−.007	.069	−.005	.001	15.369	15.582	−.007	.013	−.053	.184	−.009	.011
TG	7.162	7.223	.003	.008	.019	.089	−.005	.000	15.783	15.966	−.002	.015	−.025	.179	−.011	.007
TI	7.009	7.073	.003	.008	.020	.093	−.006	.000	16.361	16.552	−.002	.016	−.049	.164	−.010	.008
UR	5.589	5.704	−.005	.005	−.024	.109	−.002	.008	13.530	13.745	−.017	.001	−.128	.125	−.002	.016
VD	7.882	7.919	.003	.006	.010	.052	−.004	.000	17.169	17.351	−.004	.014	−.071	.132	−.009	.010
VS	7.472	7.550	.001	.008	−.032	.056	−.005	.003	16.060	16.269	−.005	.016	−.082	.152	−.013	.008

Note: Results are based on 84 months from 01/2012 to 12/2018 including the canton-specific reintroduction dates of the moratorium for SP, see Figure ??. The notations "Lower" and "Upper" refer to the lower and upper bounds of the 95%-confidence interval.
Table 10 Confidence intervals for the regression results presented in Table 3, main document.

I. Supplementary analyses and validation tests

In the following, we provide a set of statistical diagnostics to measure the validity and performance of our models. First, we discuss the goodness-of-fit by comparing AIC values between applicable models and by analyzing model residuals. Second, we provide rationales for the interrupted time series linearity assumption. Third, we present the results of a falsification test, which we implemented on the period prior the moratorium removal. Fourth, for the moratorium reintroduction, we apply our model to a control group and compare the results with the treatment group. Finally, we present out-of-sample predictions based on the data prior moratorium removal and compare them with our model prediction.

Goodness-of-fit: In Table 11, we present the AIC values when fitting the observed data for the response variables measuring the number of doctors billing in free practice N_t and health costs from doctors C_t to selected distributions. Considering the relevant observation periods for the removal and reintroduction of the moratorium (01/2007–06/2013 and 01/2012–12/2018), we separately assess the case of SP and GP. For the number of doctors billing in free practice, we report the goodness-of-fit on the negative binomial and Poisson distributions, while we present results for log-normal and Weibull distributions fitted on the health costs. In our procedure, we have also considered the binomial and geometric distributions for N_t and the exponential, normal and Gamma distributions for C_t. In all cases, we find that the negative binomial distribution best fits the number of doctors in terms of AIC, while the log-normal distribution outperforms the Weibull distribution and is best suited for health costs.

The analysis of residuals is a relevant statistical tool for evaluating the goodness-of-fit of the interrupted time series model [2]. This method consists in identifying structure, i.e., patterns, in the model residuals. The presence of a pattern is a strong signal for a potential model misspecification or a missing variable. In other words, it indicates that part of the dependent variable behavior is captured by the model error and thus remains unexplained. In contrast, when no structure is observed, we can be confident on the model specification and can reasonably assume that no fundamental covariates are missing in the model. In Figure 3, we present the residuals for the number of and health costs from SP around the removal of the moratorium in GE and ZH. For the presented figures, visual inspection confirms that no clear pattern is observed.

Linearity assumption: In the case of an interrupted time series model, the most important assumption to satisfy is the linearity assumption [3]. When a linear trend exists, it becomes straightforward to isolate the intervention and predict the counter factual. As recommended by [4], we verify such assumption by visual inspection of the data and of the residuals, i.e., as provided above. In Figure 4, we present the raw data for the number of SP and the health costs from SP for GE and ZH. As displayed in the figure, visual inspection indicates a linear trend in both the

	Removal of the national moratorium								Reintroduction of moratoriums			
	N_t of SP		C_t from SP		N_t of GP		C_t from GP		N_t of SP		C_t from SP	
	NB	P	LN	W	NB	P	LN	W	NB	P	LN	W
BE	994	1 360	2 613	2 630	930	1 042	2 587	2 618	1 161	2 426	2 863	2 870
GE	942	1 226	2 585	2 595	862	934	2 480	2 488	1 065	1 585	2 843	2 842
TI	923	1 302	2 439	2 462	874	1 073	2 388	2 405	1 069	2 004	2 753	2 758
VD	1 025	1 691	2 605	2 613	956	1 241	2 532	2 552	1 168	2 557	2 868	2 875
VS	1 014	1 956	2 425	2 437	943	1 372	2 389	2 406	1 152	2 903	2 652	2 675
ZH	1 029	1 452	2 695	2 716	974	1 140	2 678	n.a.	n.a.	n.a.	n.a.	n.a.

Note: The observation period for study of the removal of the national moratorium is from 01/2007 to 06/2013 for SP and GP. The reintroduction of cantonal moratoriums only concerns SP and is studied on the period from 01/2012 to 12/2018. The columns "N_t" and "C_t" refer to the number of and the health costs from SP respectively GP. The abbreviations "NB" and "P" mean model fits with a negative binomial respectively a Poisson distribution while "LN" and "W" denote fits with a log-normal and a Weibull distribution respectively. "n.a." stands for not applicable. In fact, in the canton ZH, no moratorium has been reintroduced, see Figure 1, main document.

Table 11 Goodness-of-fit AIC values for the number of and the health costs from SP and GP in selected cantons.

Seasonal component	Number of billing GP	SP	Health costs GP	SP
Additive	1.98	2.42	2.12	2.31
Multiplicative	1.99	2.44	2.20	2.37

Table 12 Additive and multiplicative autocorrelation results for the seasonal components in the number of billing and the health costs for GP and SP separately.

number of SP and health costs from SP. Further, we remark that the linear trend post-moratorium removal deviates significantly to the linear trend pre-moratorium removal when focusing on the number of SP. This is less the case for the health costs from SP. Finally, let us mention that the methodology applied, and our conclusion on the applicability of interrupted time series model, are identical to Figure 3 in [4].

Falsification test: In the following, we apply the falsification test to verify alternative dates on the model applied for the removal of the national moratorium for SP. In particular, this approach allows us to measure if changes in slope are related to the intervention or could be observed also at other dates [5]. For this purpose, we select the alternative dates 06/2008 and 06/2010 since both ensure having enough data points before and after the hypothetical intervention. In Table 13, we present the results of this supplementary analysis for the number of SP and health costs from SP over the 60 months period prior the moratorium removal date. For illustration purposes, we show the results for the five most populated cantons (AG, BE,

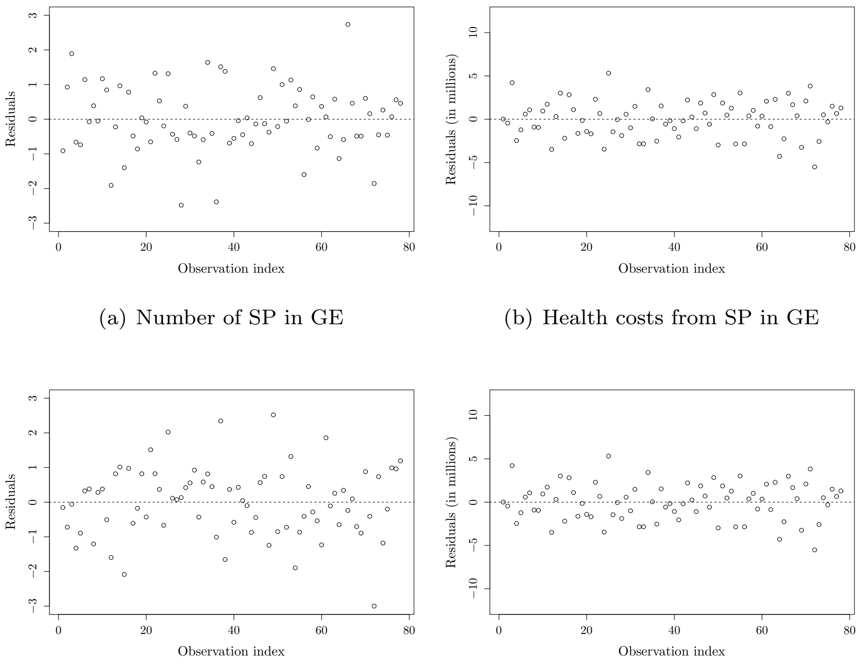

(a) Number of SP in GE (b) Health costs from SP in GE

(c) Number of SP in ZH (d) Health costs from SP in ZH

Figure 3 Residuals for the number of and health costs from SP around the removal of the moratorium in GE and ZH.

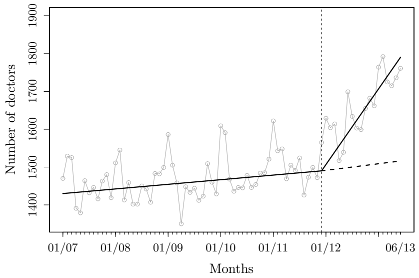

(a) Number of SP in GE

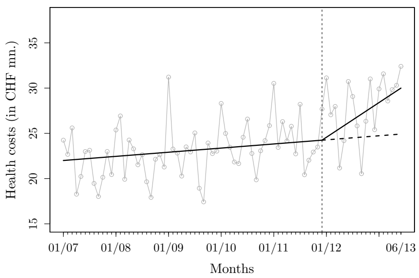

(b) Health costs from SP in GE

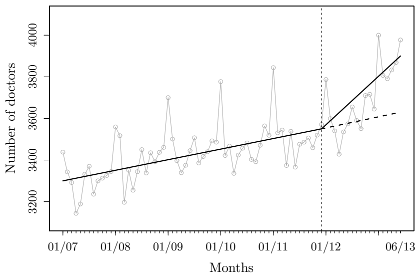

(c) Number of SP in ZH

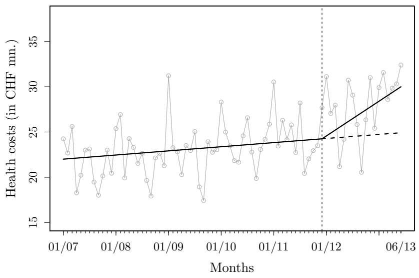

(d) Health costs from SP in ZH

Note: In each graph, the observed data points are illustrated in gray. A vertical dashed line indicates the last date before the removal of the moratorium.

Figure 4 Linearity assumption for the number of and health costs from SP around the removal of the moratorium in GE and ZH.

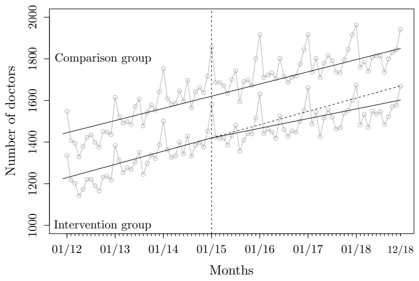

(a) Number of SP

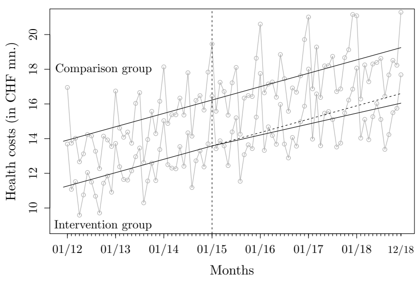

(b) Health costs from SP

Note: In each graph, the observed data points are illustrated in gray. A vertical dashed line indicates the last date before the removal of the moratorium.

Figure 5 Trend assumption in the comparison and the intervention group for the number of and health costs from SP around the removal of the moratorium in the DID model.

GE, VD and ZH). For both alternative dates, we observe that the coefficients for the moratorium removal ("Rem.") and for the interaction term ("Month $\times$ Rem.") are not statistically significant. As expected, results from this falsification test contrast with the significant factors found in Table 1 of the main article for the number of

SP billing in free practice, i.e., where we clearly observe a significant intervention effect, while they do not diverge from what we observe for the health costs from SP, i.e., where no significant intervention effect has been identified.

	Number of SP billing in free practice							Health costs from SP				
	Intercept		Month		Rem.	Month × Rem.		Intercept		Month	Rem.	Month × Rem.
Alternative date: 06/2008.												
AG	7.847	***	.021	*	.057	−.000		16.506	***	.028	.395	.017
BE	7.766	***	.018	*	.012	−.002		17.168	***	.029	.176	.007
GE	7.280	***	−.000		−.036	.010		16.925	***	.038	−.263	−.006
VD	7.706	***	.019	**	.047	.005		16.957	***	.035	.193	.004
ZH	8.121	***	.018	***	.130	−.011	*	17.801	***	.023	.117	.014
Alternative date: 06/2010.												
AG	7.907	***	.023	***	.088	−.014		16.667	***	.055	.159	−.033
BE	7.811	***	.018	***	.100	−.014	***	17.271	***	.037	.174	−.009
GE	7.295	***	.005		.101	.004		16.954	***	.018	.62	−.014
VD	7.768	***	.023	***	.089	−.003		17.064	***	.039	.447	−.030
ZH	8.162	***	.016	***	−.053	−.012		17.900	***	.036	.453	−.035

Note: Results are based on 60 months, i.e. accounting only for the 60 months before the removal of the moratorium for SP(01/2007–12/2011), see Figure 1, main document. The displayed values for the coefficients for "Month", "Rem." and "Month × Rem." are multiplied by 10. Values account for the seasonal effect. Significance levels are indicated as follows: * $p < 0.1$, ** $p < 0.05$, *** $p < 0.01$.
Table 13 Alternative dates test for the SP national moratorium removal in January 2012.

Control group: To disentangle the effect of policy under study from any other policy change or any other unobservable factor affecting the outcome variable over time, we tested our model on the control group composed by the cantons having not experienced a moratorium reintroduction (i.e. AG, AI, AR, FR, GR, JU, ZG and ZH). Such analysis is possible only for the moratorium reintroduction since, for the moratorium removal, the political intervention has been effective in all cantons. In Table 14, we present the results for the moratorium reintroduction for the cantons having not experienced reintroduction. As for the results on the treatment group (cf. Table 3, main article), we do not observe any statistically significant coefficients for the interaction term (Month × Reint.).

	Intercept		Month	Reint.		Month × Reint.
AG	16.855	***	.045	.609	**	−.012
AI	12.914	***	.028	.507	**	−.013
AR	14.370	***	.075	.473	**	−.045
FR	16.269	***	.074	.159	**	−.043
GR	15.477	***	.058	.658	**	−.030
JU	14.717	***	.100	.510	**	−.055
ZG	15.361	***	.046	.724	**	−.018
ZH	18.042	***	.028	.792	**	.005

Note: Results are based on 84 months from 01/2012 to 12/2018 including the fictive reintroduction date of 07/2013 on the moratorium for SP. The displayed values for the 95%-confidence interval for "Month", "Reint." and "Month × Reint." are multiplied by 10. Values account for the seasonal effect. Significance levels are indicated as follows: * $p < 0.1$, ** $p < 0.05$, *** $p < 0.01$.
Table 14 Regression results for the SP moratorium reintroduction for the cantons having not experienced reintroduction.

Out-of-sample test: The out-of-sample test is a powerful tool to assess the forecasting accuracy of a model [6]. In our setup, we use this approach to compare whether our model results (model) post moratorium removal diverge from the forecast on the same period (out-of-sample) obtained from fitting the model only on the 60 months prior intervention. In doing so, we bring further evidence for the results presented in the main part of the manuscript that rely on p-values. In Figure 6, we present the model and the out-of-sample predictions for the number of and health costs from SP for the moratorium removal in GE and ZH. Considering the number of SP in GE and ZH in the period post moratorium removal, we note that the confidence intervals of the model predictions diverge from the ones of the out-of-sample predictions. This confirms that the removal has led to a significant increase in the number of SP. We conclude the opposite when focusing on the health costs from SP in GE and ZH since the confidence intervals of the model and the out-of-sample predictions intersect on the post-intervention period.

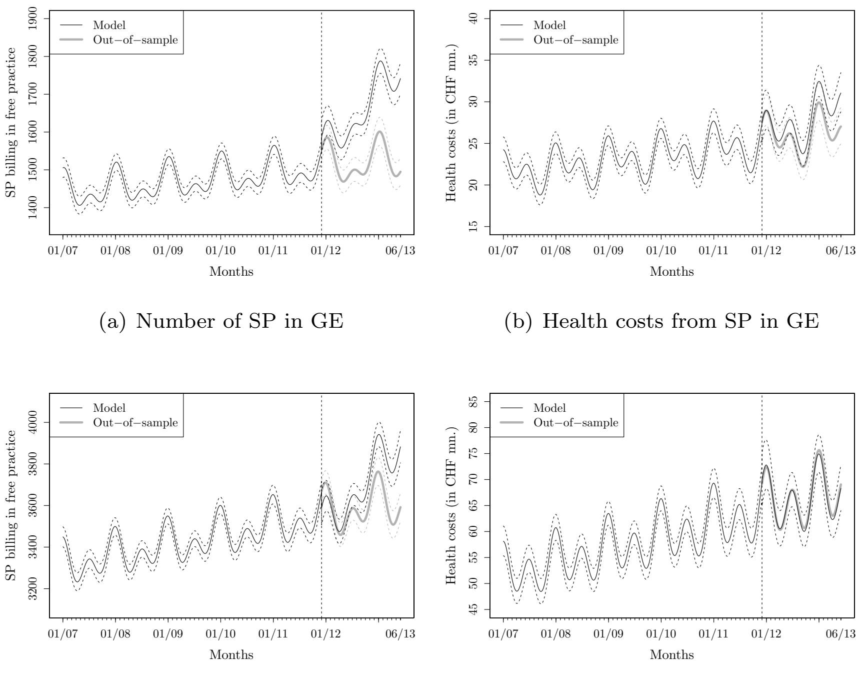

(a) Number of SP in GE (b) Health costs from SP in GE

(c) Number of SP in ZH (d) Health costs from SP in ZH

Note: In each graph, the plain curve reports the fit of the regression model and two dashed curves indicate the 95%-confidence interval. The grey curve reports the out-of-sample prediction obtained from fitting the regression model on the period from 01/07 to 12/11. A vertical dashed line indicates the last date before the removal of the moratorium.

Figure 6 Model and out-of-sample predictions for the number of and health costs from SP around the removal of the moratorium in GE and ZH.

Finally, Tables 18 and 19 present the p-values for the policy intervention and the interaction effect coefficients for both the moratorium removal and reintroduction when using the Bonferroni method. In doing so, we account for multiple testing. Results are only reported for the intervention and interaction variables. The results after correction are similar to the original ones (cf. Tables 1 and 3)

	Number of SP billing in free practice				Health costs from SP			
	Rem.		Month × Rem.		Rem.		Month × Rem.	
	Initial	Adjusted	Initial	Adjusted	Initial	Adjusted	Initial	Adjusted
AG	0.087	1.000	0.000	0.002	0.113	1.000	0.079	1.000
AI	0.319	1.000	0.643	1.000	0.259	1.000	0.175	1.000
AR	0.007	0.179	0.309	1.000	0.431	1.000	0.432	1.000
BE	0.049	1.000	0.009	0.229	0.009	0.242	0.374	1.000
BL	0.159	1.000	0.019	0.507	0.837	1.000	0.020	0.515
BS	0.034	0.889	0.000	0.000	0.697	1.000	0.545	1.000
FR	0.064	1.000	0.000	0.002	0.888	1.000	0.778	1.000
GE	0.058	1.000	0.000	0.000	0.227	1.000	0.206	1.000
GL	0.671	1.000	0.145	1.000	0.324	1.000	0.011	0.296
GR	0.001	0.025	0.000	0.000	0.382	1.000	0.780	1.000
JU	0.920	1.000	0.053	1.000	0.669	1.000	0.037	0.954
LU	0.354	1.000	0.000	0.000	0.000	0.007	0.650	1.000
NE	0.772	1.000	0.054	1.000	0.278	1.000	0.786	1.000
NW	0.573	1.000	0.634	1.000	0.665	1.000	0.250	1.000
OW	0.962	1.000	0.344	1.000	0.014	0.354	0.344	1.000
SG	0.490	1.000	0.001	0.038	0.084	1.000	0.836	1.000
SH	0.111	1.000	0.002	0.049	0.054	1.000	0.002	0.055
SO	0.036	0.941	0.003	0.072	0.338	1.000	0.940	1.000
SZ	0.286	1.000	0.036	0.934	0.088	1.000	0.285	1.000
TG	0.425	1.000	0.162	1.000	0.103	1.000	0.798	1.000
TI	0.656	1.000	0.022	0.580	0.363	1.000	0.340	1.000
UR	0.511	1.000	0.012	0.308	0.948	1.000	0.000	0.000
VD	0.932	1.000	0.011	0.277	0.074	1.000	0.801	1.000
VS	0.784	1.000	0.317	1.000	0.668	1.000	0.690	1.000
ZG	0.771	1.000	0.009	0.223	0.076	1.000	0.491	1.000
ZH	0.096	1.000	0.000	0.000	0.168	1.000	0.074	1.000

Table 15 p-values by cantons for the SP national moratorium removal in January 2012. The displayed values for the "adjusted" p-values are for the Bonferroni multiple testing adjustment method.

	Number of SP billing in free practice				Health costs from SP			
	Reint.		Month × Reint.		Reint.		Month × Reint.	
	Initial	Adjusted	Initial	Adjusted	Initial	Adjusted	Initial	Adjusted
BE	0.815	1.000	0.000	0.000	0.620	1.000	0.064	1.000
BL	0.001	0.014	0.000	0.000	0.992	1.000	0.000	0.001
BS	0.068	1.000	0.000	0.000	0.771	1.000	0.034	0.621
GE	0.840	1.000	0.000	0.000	0.235	1.000	0.047	0.853
GL	0.000	0.000	0.003	0.050	0.002	0.027	0.024	0.427
LU	0.004	0.067	0.019	0.343	0.282	1.000	0.937	1.000
NE	0.013	0.228	0.279	1.000	0.007	0.131	0.572	1.000
NW	0.001	0.013	0.003	0.046	0.166	1.000	0.109	1.000
OW	0.074	1.000	0.928	1.000	0.614	1.000	0.365	1.000
SG	0.000	0.000	0.000	0.003	0.000	0.000	0.211	1.000
SH	0.034	0.610	0.003	0.058	0.068	1.000	0.021	0.370
SO	0.000	0.007	0.003	0.050	0.058	1.000	0.070	1.000
SZ	0.045	0.813	0.013	0.231	0.066	1.000	0.586	1.000
TG	0.002	0.028	0.012	0.213	0.028	0.511	0.298	1.000
TI	0.000	0.000	0.000	0.008	0.047	0.840	0.511	1.000
UR	0.134	1.000	0.064	1.000	0.919	1.000	0.001	0.010
VD	0.010	0.175	0.110	1.000	0.292	1.000	0.900	1.000
VS	0.433	1.000	0.421	1.000	0.401	1.000	0.420	1.000

Table 16 p-values by cantons for the SP national moratorium reintroduction. The displayed values for the "adjusted" p-values are for the Bonferroni multiple testing adjustment method.

Acknowledgements

All authors thank Stéphane Luyet for his invaluable support. Further, the authors are thankful for the comments on earlier versions of this manuscript from participants of the Swiss Health Economics Workshop (Lucerne, Sept. 2019). The provision of the aggregated SASIS data for this research is gratefully acknowledged.

Funding

The authors acknowledge financial support from the Swiss National Science Foundation (grant no. 100018_169662 and grant no. CRSII5_180350).

Availability of data and materials
The data used in this article is not publicly available as it is the property of private organizations (Swiss health insurers). Link to the data: https://www.sasis.ch/. The data can be requested from this organization. No administrative permissions were required to obtain the SASIS data.

Ethics approval and consent to participate
Ethics approval and consent to participate on research with macro-level data is not required, in Switzerland (https://submissions.swissethics.ch/en/). No administrative permissions were required to obtain the SASIS data.

Competing interests
The authors declare that they have no competing interests.

Consent for publication
Not applicable.

Authors' contributions
All authors made substantial contributions to the conception and design of the work, the analysis and interpretation of data and drafted and revised the work. All authors have read and approved the manuscript.

Author details
[1]Department of Actuarial Science, University of Lausanne, Chamberonne - Extranef, 1015 Lausanne, Switzerland. [2]Department of Political Studies, University of Lausanne, Géopolis, 1015 Lausanne, Switzerland. [3]Swiss Finance Institute, University of Lausanne, Chamberonne - Extranef, 1015 Lausanne, Switzerland.

References
1. Stock, J.H., Watson, M.W.: Introduction to Econometrics, 3rd edn. Pearson, New York (2015)
2. Wagner III, J.A., Rubin, P.A., Callahan, T.J.: Incentive payment and nonmanagerial productivity: An interrupted time series analysis of magnitude and trend. Organizational Behavior and Human Decision Processes **42**(1), 47–74 (1988)
3. Wagner, A.K., Soumerai, S.B., Zhang, F., Ross-Degnan, D.: Segmented regression analysis of interrupted time series studies in medication use research. Journal of clinical pharmacy and therapeutics **27**(4), 299–309 (2002)
4. Bernal, J.L., Cummins, S., Gasparrini, A.: Interrupted Time Series Regression for the Evaluation of Public Health Interventions: A Tutorial. International Journal of Epidemiology **46**(1), 348–355 (2017). doi:10.1093/ije/dyw098
5. Chamlin, M.B., Burek, M.W., Cochran, J.K.: Welfare policy as social control: A specific test of the piven and cloward thesis. Criminal Justice Policy Review **18**(2), 132–152 (2007)
6. Tashman, L.J.: Out-of-sample tests of forecasting accuracy: an analysis and review. International journal of forecasting **16**(4), 437–450 (2000)
